# Fixing the Gap: Gendered Use of the Right to Choose Repair

**DOI:** 10.1007/s10603-026-09626-4

**Published:** 2026-07-23

**Authors:** Joasia Luzak, Vanessa Mak, Helen Pluut, Joerg Weber

**Affiliations:** 1 Law School, University of Exeter, Exeter, United Kingdom; 2 Leiden University, Leiden, Netherlands; 3 Department of Economics, University of Exeter, Exeter, United Kingdom

**Keywords:** Gender equity, Consumer protection, Repair, Enforcement barriers, Institutional design

## Abstract

The “right to choose repair” in European consumer law is central to sustainable consumption and consumer protection, yet its effectiveness depends on equitable access and enforcement across all consumer groups. While (consumer) law assumes that gender-neutral legal frameworks ensure equal protection, this premise remains empirically untested in the context of repair rights. This paper presents the first empirical study of gender’s role in enforcing the right to choose repair. Based on representative survey data from 3,141 consumers in the United Kingdom and the Netherlands, gender-based differences in adherence to the right to choose repair and factors shaping willingness to enforce it are analysed. Results show that women face distinct barriers, including acting less consistently with legal rights and greater sensitivity to procedural obstacles, financial costs, and conflict. The extent and significance of these gender-based barriers vary between national contexts, pointing to targeted policy responses that address gendered patterns in consumer law enforcement.

## Applying the Gender Lens to the Right to Choose Repair

This paper presents the first empirical investigation into how gender shapes consumer behaviour in the enforcement of the right to choose repair. This research serves two critical purposes. First, it responds to calls from gender studies for a critical re-evaluation of ostensibly neutral legal systems (Albiston et al., 2002; Schmitz, 2013). We examine whether current consumer law ensures equal protection to all, providing evidence for re-evaluating its suitability for and effectiveness in achieving gender equity. Second, our findings contribute important insights into how best to design consumer protection policies that function equitably. If gender-based barriers to adherence to and enforcement of rights persist, then consumer protection frameworks – despite being designed to be gender-neutral – may systematically disadvantage women. This is especially timely, as European policymakers continue to revise the right to choose repair to promote sustainable consumption (Spinaci, 2023; Walsh, 2023).

Although a substantial body of literature in consumer research and marketing has explored how gender shapes consumer preferences and behaviours, legal scholarship has largely failed to adopt gender as an analytical lens in the context of consumer law. The European legal benchmark for assessing the application of consumer rights remains that of the “average consumer,” defined as a person who is “reasonably well-informed and reasonably observant and circumspect” (*Gut Springenheide GmbH, Rudolf Tusky v. Oberkreisdirektor des Kreises Steinfurt – Amt für Lebensmittelüberwachung* (case C-210/96) EU:C:^1998^:369.; *Árpád Kásler, Hajnalka Káslerné Rábai v. OTP Jelzálogbank Zrt* (case C-26/13) EU:C:^2014^:282; recital 18 of the Directive 2005/29/EC concerning unfair business-to-consumer commercial practices in the internal market (2005) OJ L 149/22). This is a normative benchmark for assessment of consumer protection, mostly in the context of unfair contract terms or unfair commercial practices, and national courts are free to refer to empirical evidence of consumer confusion to substantiate it (Mak, 2011; *Gut Springenheide*; see also *Compass Banca SpA v Autorità Garante della Concorrenza e del Mercato (AGCM)* (case C-646/22) EU:C:^2024^:957). It is gender-neutral in theory, assuming that all consumers engage with their rights identically. However, given the longstanding male-default bias embedded in various legal frameworks (Epker, 2023; Perez, 2019), there is a real concern that the “average consumer” in practice reflects the characteristics of an average man. That concern is strengthened by the entrenched practice in national private laws to refer to “reasonable man" benchmarks in contract and tort law, which has only recently started to shift towards gender-neutral terms (see McCaughran, 2011 and Stern, 2024, on “the man on the Clapham Omnibus”’ in English contract and tort law; Smits, 2014 on the disappearance of Roman law’s *bonus pater familias* from French and Dutch private law; Lima Rego, 2025 on *bonus pater familias* benchmark in Portuguese private law). Therefore, the normative benchmark of an “average consumer” in consumer contract law has historically been approached from a male perspective and there is a lingering suspicion that this approach affects the practical application of rights until this day – systematically disadvantaging women and hindering their access to consumer rights. Such barriers would undermine the very purpose of consumer protection.

The formally neutral approach of European consumer law, which disregards the individual characteristics of consumers, has come under increasing criticism. Scholars argue that the “average consumer” benchmark fails to capture the diversity of consumer experiences (Mak, 2011; Ouyang, 2024). Recent research has proposed moving beyond this homogeneous concept towards frameworks that recognise how consumer behaviour is shaped by social and community contexts (Ouyang, 2024). While other areas of law – such as employment law – have long incorporated gendered perspectives (Elson, 1999), consumer law continues to be framed in gender-neutral terms. Considering the historical origins of the “average consumer” benchmark in contract law as a male-oriented standard, there is a real concern that differences between men and women are insufficiently taken into account by courts and legislators when applying this benchmark. Gendered patterns in consumer behaviour likely interact with adherence to legal rights and enforcement capacity in ways that lead to unequal outcomes, thereby undermining one of the core objectives of consumer law: to rebalance the power asymmetry between consumers and businesses (Ramsay, 2012).

The consequences of this oversight are already visible. In some cases, women are more susceptible to unfair commercial practices (Duivenvoorde, 2015), yet men are more likely to report misleading claims to authorities (Advertising Standards Authority, 2016). These findings reveal gendered effects in both vulnerability to deception and the enforcement of protection. Still, empirical findings of this kind have not led to a reassessment of consumer rights. Although contemporary consumer law research, in particular in the context of digital markets, has begun reassessing inequality and the vulnerability of specific consumer groups (Grochowski, 2024; Mak, 2024), the role of gender has not been specifically addressed. Given that the lack of effective enforcement acts as a barrier to achieving consumer protection aims (Benöhr, 2013; Pavillon, 2020), it becomes essential to understand how gender affects the progression from experiencing a problem to pursuing legal remedies.

In the meantime, consumer protection policies in general are increasingly recognising the value of behavioural and demographic insights (Helleringer & Sibony, 2017; Reisch & Zhao, 2017). Notably, international recognition of gender-related effects on consumer protection is also emerging. For example, the UN Conference on Trade and Development has observed that “information and advertising are often not presented in a gender-neutral way,” and that consumer protection should consider how “consumer education contributes to women's knowledge and bargaining power” (UN Trade & Development, 2022). Our research starts from the premise that empirical research can also help law- and policymakers in the EU gain a better understanding of gender-specific patterns with a view to improving the effectiveness of consumer rights.

Although empirical research on how gender affects consumers’ acting on their legal rights and enforcement behaviour remains scarce, a rich body of evidence from psychology and economics provides a strong foundation for expecting gender-based differences in consumer rights enforcement. Documented patterns suggest that procedural barriers – such as the need for confrontation, financial uncertainty, or significant time investment – may disproportionately discourage women from asserting their consumer rights (see, for example, Bear et al., 2011; Charness & Gneezy, 2012; Craig & Mullan, 2011; Holt & DeVore, 2005). These gendered patterns in adherence to and enforcement of legal rights may arise from differences in consumers’ legal literacy of their legal rights, both in terms of substantive aspects (such as the knowledge that they have a right to choose repair) and procedural aspects (such as how to request a repair or file a complaint). Limited understanding of either aspect can reinforce unequal access to justice and hinder effective redress in consumer sales markets (Hesselink, 2023).

To address both substantive and procedural aspects, we examine whether consumers act on their legal right to choose repair and enforce it in the UK and the Netherlands. These two countries share comparable quality of life, similar gender pay gaps (14.2% and 14.4%, respectively, Deloitte, 2023; Office for National Statistics, 2023) high women's employment rates (68% and 72.2%; EURopean Employment Services, 2023; Office for National Statistics, 2024), active consumer and market authorities, robust consumer advocacy sectors, and media that regularly cover consumer rights and the problems consumers face when seeking redress (Phillips, 2022; Radar, 2023). Both countries also share harmonised legal frameworks: all EU Member States – including the UK at the time – were required to ensure a minimum level of consumer protection in sales contracts, including the adoption of the right to choose repair for non-conforming goods, through the implementation of the Directive, 1999/44/EC on certain aspects of the sale of consumer goods and associated guarantees (1999) OJ L 171/12 (“old Consumer Sales Directive”). While the Directive (EU) 2019/771 on certain aspects concerning contracts for the sale of goods (2019) OJ L 136/28 (“new Consumer Sales Directive”) introduced some changes, these were implemented only in the Netherlands, as the UK had exited the EU by the time of adoption. Nonetheless, amendments to the right to choose repair were minimal and have been duly considered in our empirical study design. This enables meaningful analysis of gender effects across different institutional and cultural contexts.

Our research aims to strengthen the consumer protection framework for the right to choose repair and to address consumer enforcement issues in the EU. The findings contribute to the ongoing re-evaluation of the consumer repair framework, which recently led to the adoption of the Directive (EU) 2024/1799 on common rules promoting the repair of goods (2024) OJ L 2024/1799. As European policymakers continue refining this right, our survey results can at the very least inform expectations regarding the impact of new regulations. Furthermore, by uncovering gender-based inequalities, our study may support the development of targeted public information campaigns to ensure that the newly revised right to choose repair is effectively promoted and accessible to all.

We pursue these objectives against the background of prior empirical studies on gender dynamics in consumer sales law, highlighting both established insights and knowledge gaps regarding how gender affects adherence to and willingness to enforce consumer rights. The following sections introduce the consumer protection framework for the right to choose repair, along with consumer enforcement issues in the EU, UK, and the Netherlands. We then present our research questions, grounded in prior empirical scholarship, followed by a detailed outline of our study design and methodology, an overview of our empirical results, and a critical discussion. We conclude with policy recommendations based on our findings.

## Legal Framework on Repair of Consumer Goods

The right to choose repair is largely comparable in the Netherlands and the UK, as both countries closely align with the minimum harmonisation standard established by the EU legislator under the old Consumer Sales Directive. In the Netherlands, the Directive was implemented in Book 7 of the Burgerlijk Wetboek (Dutch Civil Code (DCC)). In the UK, it was initially implemented in Part 5 A of the Sale of Goods Act (SGA) 1979, later replaced by the Consumer Rights Act (CRA) 2015.

The essential elements of the right to choose repair are as follows: consumers are entitled to request repair from the seller if goods lack conformity at the time of delivery; the repair must be provided free of charge; and consumers must have a free choice between repair and the alternative remedy of replacement. The lack of conformity results from the goods not meeting either objective or subjective requirements, such as fit for purpose, agreed-upon quality and quantity, and correspondence to the sample or model. These principles are pursuant to Article 3 of the old Consumer Sales Directive, which was implemented in the UK under Sect. 23 CRA, and pursuant to Articles 13(2) and 14 of the new Consumer Sales Directive transposed into Article 7:21 DCC in the Netherlands.

One notable difference between the two jurisdictions concerns the time period for reversing the burden of proof. In the Netherlands, this period has been extended to one year, pursuant to Article 7:18(2) DCC, implementing Article 11(1) of the new Consumer Sales Directive. The UK retains the earlier period of six months, as set out in Sect. 19(14) CRA, which follows Article 5(3) of the old Consumer Sales Directive. Our study design accounts for these differences in the burden of proof.

In June 2024, the European Commission adopted a “Right to Repair” Directive, which must be implemented into the national laws of the EU Member States by 31 July 2026. It amends certain provisions of the new Consumer Sales Directive, without, however, altering the core characteristics of the right to choose repair as outlined above. Nevertheless, our study was conducted in 2023 and is based solely on the legal framework in force at that time.

## (The Lack of) Existing Research on Gender in Consumer Sales Law and Consumer Behaviour

While consumer sales law has been widely studied, the influence of sociodemographic factors – particularly gender – on how consumers engage with their rights remains underexplored. This gap persists despite growing recognition that effective consumer protection increasingly requires behavioural insights to understand how consumers actually exercise their rights (Helleringer & Sibony, 2017; Reisch & Zhao, 2017), and despite calls to move beyond traditional legal concepts that may not serve all consumers equally (Howells & Weatherill, 2017).

Prior research has examined gendered patterns in consumer transactions, including purchasing decisions (Meyers-Levy & Loken, 2015), the role of guilt in such decision-making (Kayal et al., 2017), and the incidence of fraud by sellers (Perodaud et al., 2022). These studies suggest that both individual and contextual factors shape how women and men interact with consumer protection. Insights from psychology and economics provide strong theoretical foundations for expecting gender differences in the consumer rights’ enforcement. These differences reflect deeper theoretical perspectives, which attribute gendered patterns to a combination of evolved dispositions and socially constructed role expectations (Eagly & Wood, 1999; Wood & Eagly, 2012).

Studies consistently demonstrate that women display more risk-averse behaviour than men in financial decision-making contexts (Charness & Gneezy, 2012; Croson & Gneezy, 2009). This tendency extends to investment decisions, where women adopt more conservative portfolio strategies (Bajtelsmit & Bernasek, 1996), and to consumer financial behaviour more broadly, where they show greater sensitivity to costs and financial risks (Wagner & Walstad, 2023). Biological factors may contribute to these patterns, with research linking testosterone levels to financial risk preferences (Apicella et al., 2008). Evidence from mutual fund investment decisions confirms systematic gender differences in financial risk-taking (Dwyer et al., 2002), while experimental studies reveal that women tend to avoid competitive situations even when equally capable (Niederle & Vesterlund, 2007).

Gender differences also emerge in conflict and complaint behaviour. Research shows that women are generally more conflict-avoidant than men, preferring collaborative over competitive approaches to dispute resolution (Holt & DeVore, 2005). In political contexts, women exhibit higher levels of conflict avoidance and are less likely to engage with disagreeable content (Coffé & Bolzendahl, 2017). These tendencies extend to consumer contexts, where women report greater anxiety and discomfort in conflict situations and a lower tolerance for disagreement (Bear et al., 2011). Such conflict-avoidant patterns align with broader research on coping strategies, with meta-analyses demonstrating that women more often employ emotion-focused coping and seek social support compared to men (Tamres et al., 2002).

Time constraints may also hinder the enforcement of consumer rights. Studies show that women face greater time pressures due to a disproportionate share of unpaid household labour (ILO, 2018), creating what scholars term “time poverty” – a lack of sufficient time for personal needs and discretionary activities (Craig & Mullan, 2011). “Women dedicate on average 3.2 times more time than men to unpaid care work: 4 h and 25 min per day, against 1 h and 23 min for men.” (ILO, 2018, xxix). These constraints influence consumer decision-making, with evidence indicating that women place a higher value than men on time-saving services and convenience features (Whillans et al., 2017). “Every minute more that a woman spends on unpaid care work represents one minute less that she could be potentially spending on market-related activities (…).” (Ferrant et al., 2014, 1).

Understanding how consumers move from experiencing a problem to pursuing a legal remedy requires examining legal literacy of their legal rights. Recent research confirms that legal culture and legal consciousness, defined by scholars as the process of naming, blaming, and claiming that transforms personal troubles into legal disputes (Felstiner et al., 1981), significantly influence whether consumers pursue their rights (Fibrianti et al., 2023). Broader access-to-justice studies demonstrate systematic inequalities in the ability of different demographic groups to pursue claims (Sandefur, 2008). Previously, scholars have also observed that the existence of legal rights does not automatically translate into effective enforcement, as various systematic barriers may affect demographic groups differently (Nielson & Nelson, 2005).

Increasingly, researchers have questioned whether the “average consumer” benchmark adequately serves diverse consumer populations. Legal scholars note that the concept’s reliance on the model of a consumer as a rational market actor risks overlooking systematic differences in how different groups engage with their legal rights (Duivenvoorde, 2015; Ouyang, 2024). Research on EU consumer law has identified inconsistencies in how this benchmark is applied, both in the policymaking process (Mak, 2011) and in judicial interpretation (Luzak, 2020). This has prompted proposals for alternative frameworks, such as the “embedded consumer” approach, which recognises the influence of consumers’ social contexts on their preferences and behaviours (Ouyang, 2024). These critiques align with feminist scholarship's longstanding recognition that seemingly neutral legal frameworks may perpetuate gender inequalities (Hunter et al., 2010; Shaw & Lee, 2023), and with institutional design theory, which shows how formally neutral institutions can systematically advantage certain groups while disadvantaging others (Elson, 1999).

Although some existing research on the right to choose repair has addressed related topics – showing gender differences in environmental values and perceived skills of consumers, while evaluating their sustainability awareness, repair competence, and repair opportunism (Micklitz et al., 2022) – few studies have explicitly examined gender differences in adherence to consumer rights, complaint behaviour, or enforcement of consumer rights. For example, while the European Commission investigated consumer attitudes and collected sociodemographic data (European Commission, 2020b), its published infographics aggregate responses under the general category of “Europeans”, without differentiating between European women and men. As a result, a systematic understanding of how gender affects consumer engagement with legal rights – especially the right to choose repair – remains lacking. This study addresses that gap by presenting the first empirical investigation of gender differences in adherence to the right to choose repair and enforcement behaviour. Taken together, these two dimensions span the pursuit of claims described above: adherence captures whether consumers act consistently with their legal entitlement, while enforcement captures whether they go on to press the claim (Felstiner et al., 1981).

## Consumer Sales Law and Gender: Research Questions

Our main research question is whether and how gender shapes consumers' adherence to the right to choose repair and their willingness to enforce it. To address this question, we examine six specific dimensions of the consumer experience with this right. We investigate these dimensions through six sub-questions set out below, informed by established findings in psychology and economics concerning gender differences in risk aversion, conflict behaviour, and time constraints. These bodies of work prompt our inquiry into gendered patterns in consumers' adherence to the right to choose repair and their enforcement behaviour.

At the same time, we acknowledge that empirical research on gender differences in consumer legal contexts remains limited or entirely lacking. Consequently, several of our research questions, particularly Research Questions 1, 2 and 6, are exploratory in nature. This exploratory dimension is itself part of the study's novel contribution: applying insights from consumer research, psychology, and economics to questions of consumer legal literacy and enforcement behaviour. For the more exploratory questions, this study serves a theory-building function by identifying empirical patterns that future research can further investigate. In doing so, our research responds to calls for more inclusive approaches to consumer protection and advances understanding of how gender affects adherence to legal rights and enforcement behaviour in consumer contexts.

### Adherence to the Right to Choose Repair

The benchmark for applying consumer rights is that of an average consumer. Recognising the enduring tendency of many frameworks to assume a male default (see, for example, Epker, 2023; Perez, 2019), as well as the historical tendency of contract and tort law to focus on “good housefathers” (Smits, 2014), there are concerns that the “average consumer” benchmark may effectively mirror male norms rather than a truly gender-neutral construct. It seems plausible that the EU consumer protection framework – although formally neutral – reflects a male default and is neither perceived nor processed equally by all consumers. In other words, standardized information about consumer rights may not resonate with, or reach, women and men in the same way.

A previous study asked consumers to indicate whether they knew their legal rights (European Commission, 2020a). This study found that men were more likely than women to report awareness of passenger rights (35% vs. 28%). Although this study concerns a different context and relies on self-reported knowledge, it illustrates a potentially broader pattern of gender differences in consumers’ legal literacy. This suggests that women may be less literate of their consumer rights also in cases involving defective products, including the right to choose repair.

Any gaps in legal literacy can have practical implications. Prior research has shown that consumers often choose not to pursue dispute resolution – sometimes because the issue is resolved informally with the other party, but also due to uncertainty about their rights or how to initiate a claim (Ter Voert & Hoekstra, 2020). In cases involving non-conforming, defective goods, the first step is typically to contact the seller, who may also inform consumers of available remedies. This is not a procedural condition but a substantive one; it affects the conditions under which the right to repair itself is available, rather than the means to effectuate it. One might call it an operational understanding of the right to choose repair.

In considering whether the operational understanding of the right to choose repair is gendered, this research seeks to address the question of whether any gender is more likely to act in accordance with the right to choose repair, which would entail freely choosing between repair and replacement, and approaching the seller as the first point of contact. Such behaviour may either indicate consumers’ literacy of their right or reflect a preference for this course of action, regardless of whether they possess explicit knowledge of that legal entitlement.

- Research Question 1: To what extent is there a difference between genders in acting consistently with the legal right to choose repair, both in terms of their discretion to exercise this right (i.e., to choose between repair and replacement) and their operational understanding (e.g., of whom to approach to claim a repair)?

Given the limited empirical research on gender differences in legal literacy within consumer contexts, this research question is exploratory in nature. While studies have documented gender gaps in financial literacy (Lusardi & Mitchell, 2014), systematic research on gender differences in consumer legal literacy remains sparse. By addressing this question, the research seeks to prompt further inquiry into gendered patterns in consumer legal literacy. The empirical data collected does not exclusively demonstrate basic literacy gaps in the domain of repair rights, encompassing both an understanding of the rights themselves and the procedure for exercising them, as it may also reflect consumer preferences. Nonetheless, the observed gender differences in adherence to the right to choose repair warrant further investigation.

It should be noted that practical experience with household goods and service providers may result in a different pattern of behaviours concerning compliance with the conditions under which repair can be claimed. Consumer research suggests that men and women process information differently. According to the “selectivity hypothesis,” men tend to act as “selective processors,” focusing on bottom-line information, whereas women are more likely to be “comprehensive processors,” encoding more details and contextual cues from a message (Meyers-Levy & Loken, 2015). These cognitive processing differences also extend to information search strategies. For example, research shows that women engage in more comprehensive information searches when making consumer decisions, particularly for gift purchases (Laroche et al., 2000). Men tend to be more practical in their information search, looking for specific information on product features and capabilities, exhibiting a functional, utilitarian information search approach (Kol & Levy, 2023). However, marketing literature frequently identifies women as the main decision maker responsible for the majority of consumer purchases (Krizan et al., 2022). This greater experience with sellers, service providers, and household management may enhance their procedural compliance. This leads to questions whether either gender could be more attuned to the practical conditions of a transaction, for example, acting more consistently with the knowledge that a repair for a defective product should be provided free of charge. These matters lead to our second research question.

- Research Question 2: To what extent is there a difference between genders in acting consistently with the procedural conditions required to exercise their right to choose repair?

### Willingness to Claim the Right to Choose Repair

Granting consumers the right to choose repair without facilitating enforcement would undermine the purpose of providing effective consumer protection (Biard, 2024). While consumers’ literacy of and adherence to their rights are essential first steps towards exercising them, they do not guarantee that consumers will pursue enforcement. Identifying the factors that shape consumers’ willingness to pursue repair claims is vital to designing effective consumer protection.

Previous research has identified several factors that influence consumer enforcement behaviour and the effectiveness of consumer redress. These factors can be grouped into two categories: process-related barriers and outcome-related barriers (Slater & Higginson, 2016). Process-related barriers arise when sellers fail to facilitate an easy process for consumers to assert their rights, while outcome-related barriers reflect consumers' expectations that engaging in the complaint process will not result in a satisfactory resolution. Focusing first on process-related barriers, Slater and Higginson's (2016) study indicated that busy lifestyles may deter consumers from pursuing claims. Consumers have reported perceiving the complaint process as stressful, complicated or cumbersome (Kaur & Sharma, 2015; Slater & Higginson, 2016; Stephens & Gwinner, 1998), raising the question of whether they would be more likely to raise complaints if assured that the procedure is simple and straightforward. Furthermore, since consumers have expressed concerns about the potential length of dispute resolution (Slater & Higginson, 2016), it is worth examining whether assurances of prompt responses from sellers could influence enforcement behaviour. The legal framework supports this notion: consumers are not required to wait indefinitely and may seek alternative remedies if a seller fails to repair a product within a reasonable timeframe. In the Netherlands, Article 21(3) DCC, implementing Articles 13(2) and (4) Consumer Sales Directive, obliges sellers to provide remedies within a reasonable time and without significant inconvenience. A similar obligation is stipulated in Sect. 23(2) CRA, implementing Articles 3(3) and (5) old Consumer Sales Directive.

Overall, we expect the opportunity costs of complaining to be relatively low for most consumers, particularly given the active support of consumer organizations and the widespread media coverage of the right to choose repair in both the UK and the Netherlands (ACM ConsuWijzer, 2024; Downes, 2025; Monroe & Alcock, 2025; Phillips, 2022; van Wees, 2023). These factors are likely to encourage consumers to pursue complaints. Nonetheless, opportunity costs may be higher for women due to the time constraints they often face in daily life, as highlighted by prior research on their role as caregivers and the “part-time trap” (Güneyli et al., 2025; ILO, 2018; Samtleben & Müller, 2022). Empirical support for this expectation is provided by research on gender and time poverty, which shows that women experience greater time constraints as a result of their disproportionate responsibility for unpaid household labour, creating a form of time poverty in which individuals lack sufficient time for personal needs and discretionary activities (Craig & Mullan, 2011; ILO, 2018). Such constraints can significantly influence consumer behaviour, including the decision to take legal action. Women, in particular, may place greater value on time-saving services and convenience features (Whillans et al., 2017), and may therefore encounter more substantial barriers to engaging in complaint procedures.

Thus, while facilitating easy contact with the seller and providing a simple, convenient, quick, and comprehensible complaint procedure appears crucial for ensuring a smooth repair process, such procedures may not be equally accessible to all consumers. Gendered time constraints and societal expectations may limit women’s ability to exercise their rights, even when complaint mechanisms are designed to be user-friendly.

Moreover, research on gender differences in conflict behaviour suggests that women may be more sensitive to procedural barriers involving confrontational interactions. Studies consistently find that women exhibit greater conflict avoidance than men, characterised by heightened anxiety and discomfort in confrontational situations and reduced tolerance for disagreement (Bear et al., 2011). In consumer complaint contexts, women tend to prefer collaborative approaches to problem resolution, rather than confrontational strategies (Holt & DeVore, 2005).

Taken together, these documented differences in time constraints, preferences for convenience, and approaches to conflict support our inquiry whether the ease of initiating and navigating a repair claim is of particular importance for women. A streamlined process helps reduce the burdens of time and complexity, thereby increasing the likelihood that they will take action.

- Research Question 3: To what extent is an ease of facilitation of the repair process more important for women than for men in determining their willingness to exercise the right to choose repair?

Reluctance to pursue a claim may also stem from consumers' perception that the financial investments involved in the repair and complaint processes are not justified by the anticipated benefits of a successful repair. Slater and Higginson (2016) describe this as a process-related barrier, noting that consumers often fear that the effort required may exceed the value of resolving the dispute (Stephens & Gwinner, 1998). This effort may arise not only from the direct financial costs imposed by the seller for repairing a non-conforming good, but also from auxiliary financial costs, such as shipping the non-conforming good to the seller or paying a complaint handling fee, as well as non-monetary costs, including the time required to file a complaint. Consumer protection laws, including Article 6(1)(f) of Directive 2011/83/EU on consumer rights (2011) OJ L 304/64, shield consumers from such costs and from fees that sellers might otherwise impose as a condition for initiating a claim. However, consumers may either lack the literacy of these specific entitlements related to the right to choose repair or be unwilling to pursue them, and these effects could be gendered. Our question is therefore not whether women can afford repair costs, which would primarily reflect household income, but whether the salience of cost considerations weighs more heavily on their enforcement decisions, even where the law nominally renders the process free of charge. Our regression specifications control for household income, allowing the gender coefficient to be interpreted net of differences in earning capacity.

Several strands of research suggest that women may be more sensitive to the cost dimensions of repair claims than men. Across financial decision-making contexts, women consistently display greater risk aversion and cost sensitivity than men (Charness & Gneezy, 2012; Croson & Gneezy, 2009; Wagner & Walstad, 2023). This pattern is reinforced by structural factors: both countries analysed exhibit a persistent gender pay gap, compounded by disparities in savings and pensions (Money & Pensions Service, 2024; Wijzer in geldzaken, 2024), contributing to lower overall financial wellbeing for women (Money & Pensions Service, 2023). Taken together, these patterns suggest that the cost dimensions of the repair process, and the framing of the right to choose repair as free of charge, are likely to be more salient for women than for men in shaping their willingness to pursue a claim.

- Research Question 4: To what extent are costs related to the repair process more important for women than for men in shaping their willingness to exercise their right to choose repair?

Certain barriers to enforcement may stem from consumers' perceptions of the seller or their personal attitudes towards disputes. This brings us to what Slater and Higginson (2016) categorise as outcome-related barriers, as they reflect consumers' expectations that engaging in the complaint process will not result in a satisfactory resolution. For example, consumers may become frustrated by a perceived lack of progress in the complaint procedure, which can lead to diminished expectations and a belief that their complaints will not be taken seriously. They identified a lack of confidence in the likely outcome as a key deterrent to enforcement. This uncertainty may be exacerbated by consumers' suspicion that they themselves caused the defect – for example, due to mishandling the product. Although the right to choose repair only covers defects present at the time of delivery, consumers benefit from a reversed burden of proof for a significant period after delivery, which should, in principle, strengthen their confidence in asserting a repair claim during that timeframe.

While the gender gap has narrowed over time, research indicates that women remain generally less inclined towards assertive or confrontational behaviour than men. In conflict and complaint contexts, women report greater anxiety and discomfort, lower tolerance for disagreement (Bear et al., 2011), and a stronger preference for collaborative rather than confrontational approaches to dispute resolution (Holt & DeVore, 2005). Studies have also identified gender differences in how consumers experience the complaint process itself: women are more likely to experience feelings of guilt when engaging in such actions (Perodaud et al., 2022), which may act as a psychological barrier and reduce their willingness to engage. Taken together, these findings suggest that personal attitudes towards dispute resolution may weigh more heavily on women than on men, potentially contributing to a lower likelihood of women pursuing claims.

- Research Question 5: To what extent are women's personal attitudes towards dispute resolution more likely to deter them from asserting their right to choose repair than is the case for men?

Consumers' reluctance to enforce their rights may also arise from a preference to switch to a different brand rather than pursue a repair. We classify this attitude as an outcome-related barrier (Slater & Higginson, 2016), as consumers may view any repair outcome unfavourably once they have mentally disengaged from the product. Previous research on gender differences in brand loyalty suggests that this behaviour is influenced by a range of complex factors (Melnyk et al., 2009; Vilches-Montero et al., 2018), making it difficult to predict which gender is more likely to switch. Other studies have noted consumer interest in fashion-driven obsolescence – that is, a preference for replacing a defective product with a newer model that holds greater social value (Makov & Fitzpatrick, 2021; Micklitz et al., 2022). However, this tendency may be moderated by the endowment effect, which encourages consumers to retain their existing possessions due to the value they ascribe to their ownership (Kahneman et al., 1991).

Another reason why consumers may choose not to complain to the seller about a non-conforming product is their interest in repairing the defective product themselves or with the help of acquaintances. Previous research has documented consumers' confidence in their ability to resolve such issues independently – even when their actual skills or available support networks may not justify this confidence. For example, women engaging in do-it-yourself (DIY) repair activities often report a sense of empowerment, particularly due to the perception that they are navigating a traditionally male-dominated domain (Wolf & McQuitty, 2011). As a result, women may be more inclined than men to attempt repairs themselves. Additionally, studies have characterised women as more relationship-oriented than men (Hofstede, 2001), a trait that may lead them to rely more on social networks – such as family and friends – for assistance in repairing defective products. Conversely, previous research also suggests that women place greater value on social goals and the physical environment than men, making it plausible that they may be more inclined to pursue repair through the seller, thereby promoting more sustainable consumption behaviours (Laroche et al., 2001; Sahin et al., 2012).

With prior research pointing in opposing directions, towards alternatives that bypass the seller (brand-switching, do-it-yourself repair, reliance on social networks) on the one hand, and towards seller-provided repair (driven by social and environmental goals) on the other, no consistent directional case can be built from the available evidence. We therefore treat this as an exploratory research question, examining whether and how gender shapes the preference for alternatives to seller-provided repair without committing to a directional expectation.

- Research Question 6: To what extent is there a difference between genders in preferring alternative solutions over repairs provided by the seller?

## Method

Our methodology for answering the research questions centres on a carefully designed survey experiment, which allows us to measure both adherence to legal rights and behavioural intentions within a realistic consumer scenario.

### Design

We conducted a survey to examine how gender influences consumers’ adherence to, and willingness to enforce, their right to choose repair for non-conforming goods. Participants were presented with a scenario involving a broken dishwasher – a common household appliance for which repair is often preferred due to its size and the availability of hand-washing as a temporary alternative. The dishwasher was deliberately chosen to avoid the easy replaceability typically associated with smaller items such as toasters. Notably, the dishwasher is not a gendered product, as both men and women are equally likely to purchase and use it. Moreover, it can reasonably be assumed that most consumers would prefer professional repair services over DIY solutions in the case of such appliances. Previous surveys indicate that only 7% of consumers have attempted to repair a dishwasher themselves, and just 9% would feel comfortable doing so in the future (Ibbetson, 2021).

To focus the survey on consumers’ rights related to remedies for non-conformity – specifically, defects that existed at the time of delivery but became apparent later – the scenario clearly stated that the dishwasher was non-conforming. Participants were not required to assess conformity using legal criteria.

### Participants and Procedure

We commissioned YouGov, an international market research firm, to collect data in August 2023 through a representative online survey of consumers in the United Kingdom and the Netherlands. The survey was administered to samples drawn from YouGov's panels, which include approximately 3 million consumers in the UK and 190,000 in the Netherlands. The samples were weighted to be representative of the adult population in both countries with respect to age, gender, social class, region, and level of education, including adjustments for those without internet access. The final sample comprises 2,140 respondents from the UK and 1,001 from the Netherlands.

Summary statistics for individual characteristics are presented in Table 1. The two samples are well-balanced across gender and other demographic variables, although notable differences exist between the UK and Dutch samples, particularly in terms of age distribution, educational background, and employment status. The UK sample includes a higher proportion of respondents aged 18–24 and 35–44, and a greater percentage of low-income households. Education levels appear slightly higher in the UK sample, likely reflecting differences in the structure of the Dutch vocational education system. Employment rates are similar across both countries, though a larger share of UK respondents is retired.

**Table 1:** Summary statistics

	Full sample			UK		Netherlands	
	UK	NL	p-value	Male	Female	Male	Female
Female	0.54	0.51	0.139				
Age							
18–24	0.07	0.10	0.002	0.06	0.08	0.11	0.10
25–34	0.14	0.16	0.179	0.14	0.14	0.17	0.15
35–44	0.19	0.15	0.002	0.19	0.20	0.15	0.14
45–54	0.17	0.18	0.387	0.17	0.16	0.18	0.18
55 +	0.43	0.41	0.356	0.43	0.42	0.39	0.43
Household income (£/€)							
< 25,000	0.24	0.18	0.000	0.21	0.26	0.15	0.21
25,000–44,999	0.24	0.25	0.451	0.24	0.24	0.28	0.22
45,000–69,999	0.17	0.18	0.281	0.19	0.15	0.19	0.18
70,000–99,999	0.09	0.09	0.549	0.09	0.09	0.11	0.08
100,000	0.05	0.05	0.995	0.06	0.04	0.07	0.03
No answer	0.22	0.24	0.112	0.20	0.23	0.19	0.29
Level of education							
Below secondary education	0.03	0.05	0.000	0.03	0.03	0.07	0.04
Secondary education	0.36	0.38	0.312	0.34	0.37	0.35	0.40
Vocational training	0.20	0.24	0.022	0.20	0.20	0.25	0.23
University degree	0.41	0.33	0.000	0.42	0.41	0.33	0.33
Employment status							
Working	0.56	0.59	0.110	0.60	0.52	0.59	0.58
Unemployed	0.04	0.04	0.897	0.04	0.03	0.03	0.04
Retired	0.27	0.19	0.000	0.26	0.27	0.24	0.14
Not in labour force	0.14	0.19	0.001	0.11	0.17	0.14	0.23
Marital status							
Married/partnership	0.61	0.67	0.001	0.62	0.61	0.66	0.69
Divorced/separated	0.07	0.06	0.516	0.05	0.08	0.06	0.07
Widowed	0.04	0.03	0.305	0.03	0.05	0.02	0.04
Single	0.28	0.23	0.007	0.30	0.25	0.26	0.20
Observations	**2140**	**1001**	**3141**	**987**	**1153**	**490**	**511**

The table reports mean values of individual characteristics by country and by country-gender split. Column 3 reports p-values from independent t-tests for the equivalence of means across countries for the full sample

When disaggregating the data by gender within each country, no significant gender differences were observed in terms of age or educational attainment. However, women in both the UK and the Netherlands reported lower household incomes on average, primarily attributable to differences in employment status. In both countries, a greater proportion of women are out of the labour force, but the share of employed women is lower in the UK sample than in the Dutch sample.

### Measures

A broad set of socio-economic data was collected as part of the survey, including respondents’ age, gender, income, education, employment status, and marital status. Participants were then presented with the following scenario: “You purchased a free-standing dishwasher online. Eight months later, when you go to use the dishwasher, you press the start button, and no program starts. You try a few things, but nothing appears to work.” Following this scenario, respondents were asked a series of questions. Three questions assessed their adherence to the right to choose repair and the procedural conditions under which it applies. Another three questions examined enforcement-related behaviour, exploring factors that influence consumers' willingness to pursue repair and the potential barriers they face in doing so. The survey concluded with an open-ended question, inviting participants to share any previous experience with exercising their right to choose repair.

The composition of our adherence to the legal right index, along with the four factors influencing the willingness to enforce rights, is summarised in Table 2 and discussed in detail in the following sections.

**Table 2:** Mapping of survey questions to variable construction

Factor	Survey items used
Adherence to the legal right	Q1 + Q2 answered correctly
Adherence to procedural conditions	Q5 statements answered correctly
Ease of Facilitation	Q3(1), Q4(3), Q4(4), Q6(1), Q6(2)
Costs	Q3(2), Q4(1), Q4(2), Q4(6)
Personal Attitudes	Q3(3), Q4(5), Q6(3), Q6(4), Q6(5)
Preference for Alternative Solutions	Q3(4), Q3(5), Q3(6), Q6(6)

#### Consumers’ Adherence to the Right to Choose Repair

We developed bespoke survey questions designed to assess to what extent consumers adhere to their legal right to choose repair and are willing to comply with the conditions for exercising this right within the EU consumer protection framework. Adherence is operationalised here by examining the extent to which respondents select actions that are consistent with the legal position under consumer law. When respondents select such actions, this may indicate either that they are literate of their legal entitlement or that their behavioural preferences happen to align with the legal right. Conversely, when respondents choose actions that are inconsistent with their legal rights, this may reflect either a lack of legal literacy or a willingness to forego their legal entitlement in favour of preferred behaviours. We refer to responses consistent with the legal position as "correct" throughout, with this dual interpretation in mind. To assess the extent of adherence to legal rights and to explore potential gendered patterns in this regard, we created three survey items based on realistic post-purchase scenarios.

First, to assess the basic literacy of and adherence to the legal right to choose repair in its operational understanding, we developed two questions (Q1 and Q2) with unambiguously defined correct and incorrect responses. These form our “adherence to the legal right index”, which ranges from 0 to 2, indicating the number of correct answers given by each respondent.

Q1 assessed respondents’ basic literacy of the substantive right to choose repair or replacement. Respondents were presented with the prompt: “Thinking about the dishwasher breaking down…”, followed by the question: “Which ONE of the following statements do you think best describes your legal rights?” The response options were: (1) I can ask the seller for repair, but only if a minor repair is required; (2) I can ask the seller for a new dishwasher, but only if the defect is irreparable; (3) I can ask the seller to repair the broken dishwasher or to deliver a new dishwasher instead, whichever solution works best at that time for me; (4) None of these; or (5) Don't know. Only option (3) accurately reflects the legal position under EU law, which grants consumers the discretion to choose between repair or replacement. In the Netherlands, this right follows from Article 7:21(1) DCC; in the UK, from Sects. 19(3)-(4) and 23 CRA. Selection of option (3) was therefore coded as demonstrating correct basic legal literacy.

Q2 measured adherence to the legal right to choose repair, by checking whether respondents would choose to contact the party who bears the legal repair obligation when seeking repair. As explained above, we consider recourse to the seller a substantive, operational aspect of the right to repair, rather than a procedural one. Respondents were asked: “Who, if anyone, would you be likely to contact about the problem looking for the dishwasher to be repaired?” The response options included: (1) The seller of the dishwasher, (2) The manufacturer of the dishwasher, (3) A handyman, (4) A friend or a family member, (5) Not applicable – I would repair it myself, and (6) Not applicable – I would not try to repair it at all. Multiple selections were allowed. Responses that included the seller were coded as correct, since EU law assigns the primary responsibility for facilitating repair to the seller in both jurisdictions.

Second, to assess adherence to the legal conditions under which the right to repair may be exercised, we introduced a scenario in which the seller imposed several procedural requirements before offering a remedy. Q5 measures whether consumers would adhere to such conditions. Answers coded as correct reveal either the respondents' literacy of the right to a reversed burden of proof and the availability of repair free of charge, or their preference for such repair conditions. The sum of correct answers to Q5 forms the “adherence to procedural conditions index”, which ranges from 0 to 6. The scenario stated that the seller “informs you that before they do anything, you must take certain actions.” Respondents were then asked how likely they would be to comply with each of the following six conditions, rated on a five-point Likert scale from 1 (very likely) to 5 (very unlikely): (1) Prove that you did not damage the dishwasher yourself; (2) Prove that the dishwasher was delivered to you damaged; (3) Prove that you have the warranty for the dishwasher; (4) Pay for delivering the broken dishwasher to the seller for repair; (5) Pay for repair of the broken dishwasher; and (6) Pay for the seller sending the repaired dishwasher back to you.

Under EU consumer law, remedies must be provided free of charge, meaning that responses indicating unlikeliness for items (4) to (6) were considered correct. Similarly, items (1) and (3) are not valid legal requirements and should also be rejected by respondents with adequate legal literacy of the right to repair. The assessment of item (2) is jurisdiction-specific: in the Netherlands, the presumption of non-conformity applies for 12 months following delivery, and thus no proof is required in the presented scenario (i.e., the dishwasher broke down after eight months). In contrast, in the UK, this presumption applies only for six months, so respondents with legal literacy might reasonably accept this condition and answer our survey item with “fairly likely” or “very likely”. Again, correct answers may also reflect respondents' behavioural preferences for repair options that are consistent with legal requirements.

Together, the adherence to the legal right and adherence to procedural conditions indices offer a multidimensional operationalization of the basic legal literacy of and respondents’ willingness to adhere to the right to repair.

#### Ease of Facilitation of Repair

To assess ease of facilitation as a factor influencing consumers’ willingness to enforce their right to choose repair, we operationalized this concept through five targeted survey items designed to capture perceived simplicity of the repair process. These items collectively reflect how procedural convenience and time-related barriers impact consumers’ enforcement behaviour.

The first item (Q3, statement 1) asked respondents to consider the likelihood that ease of contacting the seller would influence their decision to request a repair. Specifically, the prompt stated: “How likely, if at all, is it that the following factors would influence your decision to ask the seller to repair the dishwasher?” with one of the response options being: “It is easy to contact the seller”. This item was rated on a five-point Likert scale ranging from “very unlikely” to “very likely.”

The second and third items (Q4, statements 3 and 4) addressed the perceived clarity and responsiveness of the complaint procedure. Respondents were asked to consider how likely they would be to file a complaint under certain conditions, with the relevant statements being: “The complaint procedure is easy and understandable” and “The seller responds quickly to complaints”. Both were rated using the same five-point scale.

The fourth and fifth items (Q6, statements 1 and 2) assessed barriers experienced after an initial complaint. In this scenario, respondents were told that two weeks had passed without a reply from the seller and were asked what might influence their decision to abandon the repair request. The relevant statements were: “The seller does not respond at all” and “Not having time during the seller's office hours to complain and insist on the repair”. These were measured on a five-point scale ranging from “strongly disagree” to “strongly agree.”

Responses to all five items were averaged to construct a composite “ease of facilitation of repair” index, where higher values indicate that ease of facilitation plays a more influential role in the respondent's decision to pursue repair. This measure captures both logistical and temporal dimensions of accessibility in the enforcement of consumer rights.

#### Costs of Repair Process

To assess cost sensitivity as a factor influencing consumers’ willingness to enforce their right to choose repair, we constructed a composite measure based on four survey items capturing perceived financial burdens associated with the repair and complaint process. These items reflect respondents’ sensitivity to both direct costs (e.g., delivery fees, charges) and cost-related expectations (e.g., whether the complaint process is perceived as free).

The first item (Q3, statement 2) asked respondents to consider whether: “The seller arranges and pays for delivering the broken dishwasher to them” would influence their decision to request a repair. Responses were rated on a five-point Likert scale, ranging from “very unlikely” to “very likely” to influence their decision.

The remaining three items were drawn from a subsequent question (Q4) on factors affecting the decision to complain to the seller. These were: “The complaint procedure is free of charge” (Q4, statement 1), “The complaint procedure involves only a small cost, for example, payment for a phone call” (Q4, statement 2), and “You know in advance that the repair of the dishwasher will be free of charge” (Q4, statement 6). Each item was rated on the same five-point Likert scale.

Responses to these four items were averaged to create a single cost sensitivity index for each respondent, with higher values indicating that financial considerations play a more significant role in their decisions to pursue repair. This index captures the extent to which perceived or anticipated costs may act as a barrier to the enforcement of consumer rights.

#### Personal Attitudes Towards Dispute Resolution

To assess personal attitudes towards dispute resolution as a factor influencing enforcement behaviour, we constructed a composite variable based on five survey items capturing respondents' confidence, self-attribution, and emotional disposition towards the dispute process. These items were designed to measure psychological barriers to pursuing repair claims.

The first item, “You might have done something that caused the dishwasher to stop working” (Q3, statement 3), reflects a sense of self-blame or insecurity about the legitimacy of the claim. It was rated on a five-point scale ranging from “very unlikely” to “very likely” to influence the respondent’s decision to pursue repair.

The second item, “You are confident that reporting a problem with the dishwasher means that the problem will be solved” (Q4, statement 5), measures the respondent's trust in a successful outcome, using the same response scale.

The remaining three items addressed attitudes after a period of waiting for a response from the seller. These were: “You do not believe the seller will be able or willing to help” (Q6, statement 3), “Expecting your complaint will not be taken seriously” (Q6, statement 4), and “Feeling guilty for complaining further” (Q6, statement 5). These were rated on a five-point scale from “strongly disagree” to “strongly agree,” capturing pessimism and emotional discomfort associated with escalating a complaint.

Responses across all five items were averaged to create a composite measure, with higher scores indicating more negative personal attitudes towards dispute resolution.

#### Preference for Alternative Solutions

To assess consumers’ preference for alternative solutions over seller-provided repair, we constructed a composite variable based on four items capturing the inclination to pursue options other than having the seller perform the repair. These items reflect the relative appeal of informal solutions (e.g., self-repair, repair by a friend) and replacement options (e.g., purchasing a newer model or switching brands) in comparison to the formal repair process.

The four items examined whether a respondent's decision would be influenced by: the ability to “repair the dishwasher yourself” (Q3, statement 4); having “a friend who says they can repair the broken dishwasher for you” (Q3, statement 5); the availability of “a newer model… that you would prefer” (Q3, statement 6), and “preferring to switch to a different brand of dishwasher” (Q6, statement 6). Responses across these four items were averaged to produce a single composite measure, with higher scores indicating a stronger preference for alternatives to seller-provided repair.

### Empirical Strategy

Our empirical approach proceeds in three stages to identify gender differences in consumers’ adherence to their legal rights and enforcement willingness. First, we conduct bivariate comparisons using *t*-tests to establish baseline gender differences within each country. Second, we estimate country-specific OLS models to examine gender effects while controlling for demographic characteristics:

1 $${Y}_{i}={\alpha}_{0}+{\alpha}_{1} Femal{e}_{i}+ {X}^{\prime}_{i\gamma} + {\varepsilon}_{i}$$

We estimate this model separately for the UK and Netherlands samples. ${Y}_{i}$ represents seven distinct outcomes: (1) adherence to the legal right index also measuring basic legal literacy (0–2 scale), (2) adherence to procedural conditions index measuring actions consistent with the legal literacy of procedural conditions (0–6 scale), (3–6) four factor scores capturing barriers to enforcement – ease of facilitation, costs, personal attitudes, and preference for alternatives (1–5 scales), and (7) willingness to enforce rights (binary). The vector ${X}_{i}$ includes controls for age, education, income, employment, and marital status.

Third, we pool the data and estimate models with interaction terms to test whether gender effects vary across institutional contexts:

2 $${Y}_{i}={\beta}_{0}+{\beta}_{1}Femal{e}_{i}+{\beta}_{2}Netherland{s}_{i} +{\beta}_{3}{\left(Female\times Netherlands\right)}_{i} + {X}^{\prime}_{i\gamma }+ {\varepsilon}_{i}$$

In this specification, ${\beta}_{1}$ identifies the gender effect in the UK (baseline country), ${\beta}_{2}$ captures country-level differences for men, and ${\beta}_{3}$, the key parameter, tests whether the gender gap differs between countries. We report robust standard errors throughout and present effect sizes as percentage changes relative to baseline predicted values for interpretability. To make our approach accessible for a wide audience, we provide a detailed explanation of our statistical approach and guidance on interpretation of results in Appendix 1.

## Results

We now present the empirical findings from our survey of 3,141 consumers across the UK and the Netherlands. Our analysis addresses each of the six sub-questions in turn, examining patterns of gender differences and their variation across countries, in order to answer the main research question.

Throughout this section, "correct" responses refer to actions aligned with the legal position under the CSD, as defined in the Measures section. As discussed there, such responses indicate whether respondents adhere to the right to choose repair and may reflect either legal literacy of this right or behavioural preferences that happen to align with it.

### Adherence to the Right to Choose Repair

We first discuss results related to the two adherence indices. Table 3 presents the response rates for each answer, with correct responses for Q1 and Q2 indicated by a checkmark. Our analysis documents gender differences in the basic literacy of and adherence to the legal right to choose repair, with men giving correct responses more often than women. On average, consumers give 1.05 (out of 2) correct responses, but men consistently outperform women in both countries. Men give 1.03 and 1.20 correct responses in the UK and the Netherlands, respectively, while women give 0.96 and 1.12, addressing Research Question 1. These differences are statistically significant in the UK (*p* =.042), and although not significant in the Netherlands, the same directional pattern is observed (*p* =.063).

**Table 3:** Understanding right to choose repair

	UK			Netherlands		
	Male	Female	p-value	Male	Female	p-value
Q1 *Which ONE of the following statements do you think best describes your legal rights?*						
✗ I can ask the seller for repair, but only if a minor repair is required	0.05	0.05	0.772	0.10	0.09	0.667
✗ I can ask the seller for a new dishwasher, but only if the defect is irreparable	0.28	0.28	0.879	0.28	0.26	0.636
✓ I can ask the seller to repair the broken dishwasher or to deliver a new dishwasher instead, whichever solution works best at that time for me	0.41	0.40	0.646	0.48	0.45	0.383
✗ None of these/Don't know	0.26	0.28	0.423	0.15	0.20	0.041
Q2 *Who, if anyone, would you be likely to contact about the problem looking for the dishwasher to be repaired? Please select all that apply*						
✓ The seller of the dishwasher	0.62	0.56	0.006	0.72	0.66	0.036
✗ The manufacturer of the dishwasher	0.45	0.48	0.191	0.24	0.28	0.106
✗ A repairer/handyman	0.11	0.16	0.000	0.11	0.11	0.978
✗ A friend or family member/I would repair it myself	0.12	0.11	0.598	0.13	0.17	0.043
✗ I would not try to repair it at all	0.05	0.02	0.000	0.03	0.01	0.068
✗ Other	0.03	0.03	0.353	0.02	0.03	0.470
Q5 *You have decided to reach out to the seller who you originally purchased the dishwasher from and raise a complaint. They inform you that before they do anything, you must take certain actions*						
1 Prove that you did not damage the dishwasher yourself	0.31	0.29	0.337	0.28	0.33	0.077
2 Prove that the dishwasher was delivered to you damaged	0.36	0.42	0.003	0.25	0.27	0.407
3 Prove that you have the warranty for the dishwasher	0.10	0.08	0.112	0.09	0.06	0.083
4 Pay for delivering the broken dishwasher to the seller for repair	0.65	0.71	0.001	0.56	0.66	0.001
5 Pay for repair of the broken dishwasher	0.65	0.67	0.319	0.52	0.55	0.421
6 Pay for the seller sending the repaired dishwasher back to you	0.66	0.72	0.002	0.57	0.68	0.000
Adherence to the legal right index Q1 + Q2 (0–2)	**1.03**	**0.96**	**0.042**	**1.20**	**1.12**	**0.063**
Adherence to procedural conditions index Q5 (0–6)	**2.73**	**2.90**	**0.008**	**2.27**	**2.56**	**0.009**
Observations	**987**	**1153**	**2140**	**490**	**511**	**1001**

The table shows the response rates to the questions we ask to elicit consumers' understanding of the right to choose repair and the indices we create to summarise this understanding. p-values from independent t-tests for the equivalence of means are reported to test statistical significance for differences by gender within each country. Q1 and Q2 have one correct statement each, indicated by the checkmark. The adherence to the legal right index sums up the number of correct answers to these two questions

Q5 consists of six individual statements. Respondents were asked to select their agreement to a statement on a five-point scale from ‘very unlikely’ to ‘very likely’. For UK consumers, a statement is answered correctly if they choose ‘very unlikely’ or ‘fairly unlikely’ for all except statement 2, where a correct answer is ‘very likely’ or ‘fairly likely’. For NL consumers, all statements are answered correctly if ‘very unlikely’ to ‘very likely’ has been chosen. The adherence to procedural conditions index sums up the number of statements of Q5 answered correctly

The pattern under RQ1 is particularly evident regarding whom respondents would contact for repairs, which determines the first step in the complaint process. Men are significantly more likely than women to select the seller, who bears the repair obligation under the CSD. In the UK, 62% of men compared to 56% of women select the seller (*p* =.006), while in the Netherlands, 72% of men compared to 66% of women do so (*p* =.036). This consistent pattern across both countries suggests a robust gender difference in the basic literacy of and adherence to the operational aspects of the right to choose repair, reinforcing the broader pattern captured in RQ1.

However, when we turn to adherence to the procedural conditions under which the right to choose repair can be exercised, measured via our adherence to procedural conditions index, we observe a reversal of the earlier pattern. Here, women give correct responses more often than men in both countries, addressing Research Question 2. In the UK, women score 2.90 compared to men's 2.73 (*p* =.008), while in the Netherlands, women score 2.56 compared to men's 2.27 (*p* =.009).

Examining the individual components shows that the gender difference is largest in responses indicating that consumers should not have to "pay for delivering the broken dishwasher to the seller for repair" (UK: 71% of women vs 65% of men give correct responses, *p* =.001; Netherlands: 66% vs 56%, *p* =.001) and should not "pay for the seller sending the repaired dishwasher back" (UK: 72% vs 66%, *p* =.002; Netherlands: 68% vs 57%, *p* <.001). Women's responses are therefore more consistent with the free-of-charge principle underlying the right to choose repair.

While these gender patterns are consistent across countries, we observe contextual variation in overall response levels. Dutch consumers give correct responses more often on the legal-right items across gender, while UK consumers do so more often on the procedural-conditions items. These differences provide contextual insights but do not alter the gender patterns observed.

To provide further insights into these gender differences while controlling for other factors, we present results from two linear regression models, where the dependent variables are the adherence to the legal right index (Table 4) and the adherence to procedural conditions index (Table 5), respectively. This analysis confirms the observed gender patterns while controlling for other variables. Our multivariate models show that women and men consistently differ in how they engage with the right to choose repair: women's responses are less often aligned with the substantive legal right, but more often aligned with the procedural conditions required to exercise it.

**Table 4:** Adherence to the legal right index

	(1) UK	(2) NL	(3) All
Female	−0.072**	−0.068	−0.068**
	*(0.034)*	*(0.049)*	*(0.034)*
Netherlands			0.188***
			*(0.042)*
Female x Netherlands			−0.021
			*(0.058)*
Age			
18–24	−0.211***	−0.019	−0.129**
	*(0.082)*	*(0.097)*	*(0.062)*
25–34	−0.145**	−0.099	−0.117**
	*(0.062)*	*(0.083)*	*(0.050)*
35–44	−0.214***	−0.088	−0.170***
	*(0.056)*	*(0.081)*	*(0.046)*
45–54	−0.203***	−0.005	−0.124***
	*(0.057)*	*(0.078)*	*(0.046)*
Household income (£/€)			
25,000–44,999	0.079	0.142*	0.081*
	*(0.051)*	*(0.075)*	*(0.042)*
45,000–69,999	0.072	0.088	0.059
	*(0.059)*	*(0.083)*	*(0.048)*
70,000–99,999	−0.047	0.064	−0.023
	*(0.071)*	*(0.102)*	*(0.058)*
100,000	0.062	−0.013	0.017
	*(0.085)*	*(0.116)*	*(0.068)*
No answer	0.036	−0.035	0.006
	*(0.051)*	*(0.076)*	*(0.042)*
Level of education			
Secondary education	0.131	0.128	0.128*
	*(0.105)*	*(0.110)*	*(0.077)*
Vocational training	0.201*	0.113	0.173**
	*(0.108)*	*(0.114)*	*(0.079)*
University degree	0.191*	0.331***	0.231***
	*(0.106)*	*(0.112)*	*(0.078)*
Observations	**2140**	**1001**	**3141**
Adjusted R^2^	**0.029**	**0.022**	**0.032**
F-statistic	**4.677**	**2.316**	**6.120**
Prob > F	**0.000**	**0.001**	**0.000**
Baseline predicted score	**0.991**	**1.172**	**1.046**

The table shows results from OLS regressions where the dependent variable is the adherence to the legal right index (0–2). Columns 1 and 2 split the model into UK and Netherlands, respectively; column 3 reports results for the full sample including controls for country, gender, and country-gender effects (baseline category = UK male)

* *p* < 0.1, ** *p* < 0.05, *** *p* < 0.01. Robust standard errors in parentheses. Additional controls not shown for marital and employment status. Omitted baseline categories are for age: 55 +; household income: below 25,000; education: below secondary education

**Table 5:** Adherence to procedural conditions index

	(1) UK	(2) NL	(3) All
Female	0.187***	0.341***	0.190***
	*(0.063)*	*(0.112)*	*(0.063)*
Netherlands			−0.420***
			*(0.092)*
Female x Netherlands			0.107
			*(0.125)*
Age			
18–24	−1.242***	−0.765***	−1.105***
	*(0.149)*	*(0.213)*	*(0.122)*
25–34	−0.823***	−0.394**	−0.675***
	*(0.116)*	*(0.188)*	*(0.100)*
35–44	−0.761***	−0.221	−0.605***
	*(0.104)*	*(0.186)*	*(0.092)*
45–54	−0.413***	−0.329*	−0.383***
	*(0.107)*	*(0.178)*	*(0.093)*
Household income (£/€)			
25,000–44,999	0.070	0.544***	0.180**
	*(0.095)*	*(0.169)*	*(0.083)*
45,000–69,999	0.146	0.585***	0.247***
	*(0.109)*	*(0.183)*	*(0.094)*
70,000–99,999	0.494***	0.486**	0.452***
	*(0.137)*	*(0.240)*	*(0.120)*
100,000	0.129	0.679**	0.270*
	*(0.166)*	*(0.322)*	*(0.153)*
No answer	−0.113	0.283	−0.017
	*(0.094)*	*(0.173)*	*(0.084)*
Level of education			
Secondary education	0.043	0.327	0.186
	*(0.181)*	*(0.263)*	*(0.153)*
Vocational training	0.129	0.274	0.230
	*(0.187)*	*(0.270)*	*(0.158)*
University degree	0.097	0.695**	0.335**
	*(0.184)*	*(0.270)*	*(0.156)*
Observations	**2140**	**1001**	**3141**
Adjusted R^2^	**0.084**	**0.064**	**0.082**
F-statistic	**12.148**	**5.058**	**15.279**
Prob > F	**0.000**	**0.000**	**0.000**
Baseline predicted score	**2.816**	**2.472**	**2.693**

The table shows results from OLS regressions where the dependent variable is the adherence to procedural conditions index (0–6). Columns 1 and 2 split the model into UK and Netherlands, respectively; column 3 reports results for the full sample including controls for country, gender, and country-gender effects (baseline category = UK male)

* *p* < 0.1, ** *p* < 0.05, *** *p* < 0.01. Robust standard errors in parentheses. Additional controls not shown for marital and employment status. Omitted baseline categories are for age: 55 +; household income: below 25,000; education: below secondary education

For adherence to the legal right, women score approximately 7% lower than men in the UK (−0.072 ÷ 0.991 = −7.3% relative to the baseline prediction). This gender gap remains in the same direction in the Netherlands, although it does not reach statistical significance.

In contrast, women's higher adherence to procedural conditions is consistent across contexts. Women score 7% higher than men in the UK and 14% higher in the Netherlands on the practical requirements for exercising the right to choose repair. This translates into women giving correct responses on approximately 0.2–0.3 more procedural conditions than men, a practically meaningful difference when recognising when sellers are making inappropriate demands.

These contrasting gender patterns suggest that women and men develop distinct types of consumer legal literacy: women's responses align more closely with procedural conditions, while men's align more closely with the substantive legal right. The Female × Netherlands interaction terms are not statistically significant for either index, indicating that these patterns are robust across different institutional and cultural contexts.

To assess whether this procedural-conditions result depends on the composition of the index, we re-estimated it on two subsets of the Q5 items. The six statements combine demands that the consumer prove something (items 1 to 3) with demands that the consumer pay something (items 4 to 6), and these two types of demand may tap distinct constructs. We therefore split the index, retaining first the three "prove X" items and then the three "pay X" items. The gender difference is concentrated in the "pay X" items, where women's responses remain significantly more consistent with the procedural conditions than men's (Female = 0.172 in the UK and 0.274 in the Netherlands, both *p* < 0.01; Table 14 in the Appendix). In the "prove X" items the gender coefficient is small and not statistically significant in either country (Female = 0.015 in the UK and 0.067 in the Netherlands; Table 13 in the Appendix). This is consistent with how we interpret the index: the procedural advantage reflects women's greater tendency to reject improper demands to bear the costs of repair.

Across both models, age is a significant predictor, with younger consumers consistently scoring lower on both adherence indices than the baseline group aged 55 and above. Higher levels of education are associated with greater adherence to the legal right, while higher income is positively associated with greater adherence to procedural conditions.

### Factors that Influence Consumers’ Willingness to Pursue Repair

The previous section documented gender differences in adherence to the right to choose repair and to its procedural conditions. In this section, we examine which factors influence consumers' willingness to pursue repair, and which may prevent them from doing so. For this analysis, we use four composite variables comprising several statements, each of which respondents rated on a five-point Likert scale. Table 6 presents the average score for each statement by country and gender, along with *p*-values from independent *t*-tests assessing the equivalence of means by gender within each country, and Cohen's *d* as a measure of effect size. The effect sizes confirm that the gender differences, where statistically detectable, are modest: Cohen's *d* remains below the conventional threshold for a small effect (|*d*|= 0.2) for almost every comparison, with the largest single effect reaching only *d* = 0.29. These magnitudes are consistent with the regression coefficients reported in the subsections that follow.

**Table 6:** Factors that influence the right to choose repair

	UK				Netherlands			
	Male	Female	p-value	Cohen's d	Male	Female	p-value	Cohen's d
Q3 *Thinking about reaching out to the seller of the dishwasher: how likely, if at all, is it that the following factors would influence your decision to ask the seller to repair the dishwasher?*								
1 It is easy to contact the seller	3.94	4.00	0.196	−0.06	3.99	4.03	0.613	−0.03
2 The seller arranges and pays for delivering the broken dishwasher to them	3.89	3.83	0.303	0.04	3.81	3.62	0.010	0.16
3 You might have done something that caused the dishwasher to stop working	2.65	2.70	0.370	−0.04	2.58	2.45	0.057	0.12
4 You probably can repair the dishwasher yourself	2.45	2.30	0.005	0.12	2.37	2.18	0.013	0.16
5 You have a friend who says they can repair the broken dishwasher for you	2.41	2.51	0.059	−0.08	2.44	2.65	0.008	−0.17
6 There is a newer model available that you would prefer over your broken dishwasher	2.75	2.75	0.999	−0.00	3.04	2.70	0.000	0.29
Q4 *Thinking about reaching out to complain about the dishwasher: how likely, if at all, is it that the following factors would influence your decision to complain about the broken dishwasher to the seller?*								
1 The complaint procedure is free of charge	4.10	4.20	0.031	−0.09	3.83	3.85	0.753	−0.02
2 The complaint procedure involves only a small cost, for example payment for a phone call	3.53	3.64	0.037	−0.09	3.29	3.31	0.764	−0.02
3 The complaint procedure is easy and understandable	3.93	4.08	0.001	−0.15	3.62	3.65	0.685	−0.03
4 The seller responds quickly to complaints	3.89	3.98	0.062	−0.08	3.59	3.62	0.632	−0.03
5 You are confident that reporting a problem with the dishwasher means that the problem will be solved	3.94	4.08	0.002	−0.13	3.87	3.81	0.351	0.06
6 You know in advance that the repair of the dishwasher will be free of charge	4.01	4.12	0.027	−0.10	3.59	3.40	0.018	0.15
Q6 *It has been two weeks since you contacted the seller, and requested a repair for your broken dishwasher, you have not heard anything else from them. To what extent do you agree or disagree that the following factors would influence your decision to stop waiting for the repair by the seller (and either have it repaired by someone else or purchase a new dishwasher)?*								
1 The seller does not respond at all	3.36	3.35	0.873	0.01	2.95	3.00	0.594	−0.04
2 You do not have time during the seller's office hours to complain and insist on the repair	3.08	3.08	0.980	0.00	2.93	2.66	0.002	0.22
3 You do not believe the seller will be able or willing to help	3.39	3.43	0.509	−0.04	3.20	3.26	0.449	−0.06
4 You expect your complaint will not be taken seriously	3.37	3.39	0.776	−0.02	3.18	3.23	0.582	−0.04
5 You feel guilty for complaining further	2.04	2.26	0.001	−0.19	2.26	2.22	0.671	0.03
6 You prefer to switch to a different brand of dishwasher	3.28	3.15	0.038	0.11	3.23	3.16	0.391	0.06
Not applicable – It is unlikely I would stop pursuing my complaint	0.39	0.38	0.576	0.02	0.25	0.26	0.634	−0.03
Ease of Facilitation	**3.76**	**3.84**	**0.030**	**−0.09**	**3.50**	**3.50**	**0.955**	**−0.00**
Costs	**3.88**	**3.95**	**0.069**	**−0.08**	**3.63**	**3.55**	**0.114**	**0.10**
Personal Attitudes	**3.16**	**3.25**	**0.003**	**−0.13**	**3.06**	**3.03**	**0.400**	**0.05**
Preference for Alternative Solutions	**2.64**	**2.59**	**0.205**	**0.06**	**2.71**	**2.61**	**0.045**	**0.13**
Observations	**987**	**1153**	**2140**	**2140**	**490**	**511**	**1001**	**1001**

The table shows average scores to our questions on the factors that influence consumers' choice to choose repair. p-values from independent t-tests for the equivalence of means are reported to test statistical significance for differences by gender within each country. Each question asks respondents to answer six statements on a five-point scale. For Q3 and Q4, the scale ranges from ‘very unlikely’ to ‘very likely’. For Q6, the scale ranges from strongly disagree to agree; Q6 also includes a yes/no question on whether consumers would not stop pursuing a complaint

The bottom panel shows average scores of summary variables that we create to investigate factors that influence consumers' willingness to pursue repair. See main text for details

**Fig. 1:**
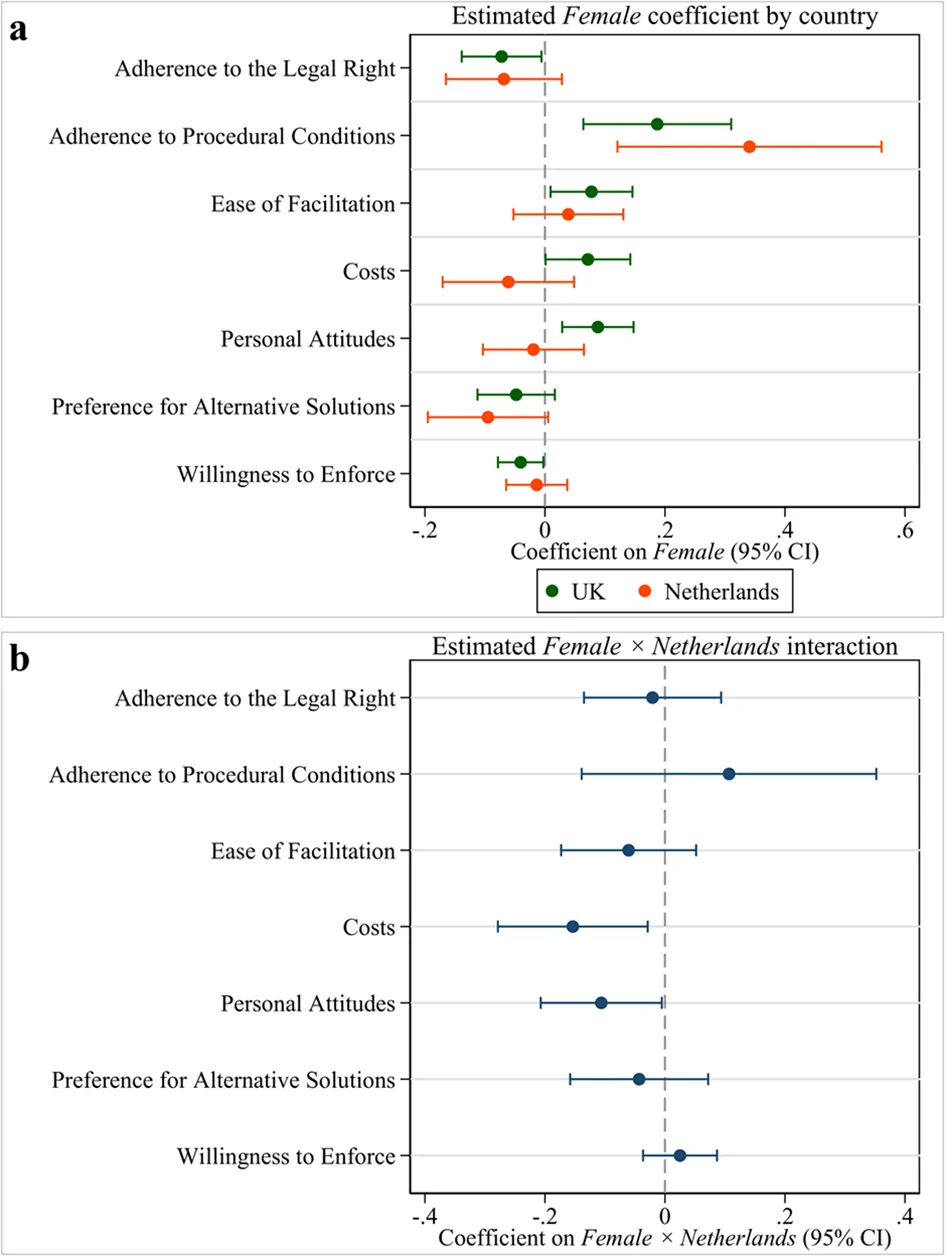
Gender differences across outcomes, by country. *Note.* Each marker is an estimated regression coefficient with its 95% confidence interval, taken from the per-outcome OLS regressions reported in the regression tables (same specifications, robust standard errors, and the full set of demographic controls). Top panel (**a**): the Female coefficient estimated separately within the UK and the Netherlands. Bottom panel (**b**): the Female × Netherlands interaction from the pooled model. Positive values indicate higher scores among women; the dashed line marks zero

Figure 1 provides a visual summary of the regression results that follow. The top panel plots the estimated Female coefficient for each outcome, separately for the UK and the Netherlands; the bottom panel plots the Female × Netherlands interaction from the pooled model. The detailed results are reported in Tables 4, 5, and7.

**Table 7:** Regression analysis of factors influencing ‘Ease of Facilitation’

	(1) UK	(2) NL	(3) All
Female	0.078**	0.039	0.081**
	*(0.035)*	*(0.047)*	*(0.035)*
Netherlands			−0.267***
			*(0.042)*
Female x Netherlands			−0.061
			*(0.057)*
Age			
18–24	0.081	0.153	0.104*
	*(0.079)*	*(0.093)*	*(0.060)*
25–34	0.003	0.121	0.043
	*(0.061)*	*(0.078)*	*(0.048)*
35–44	−0.104*	0.130*	−0.038
	*(0.056)*	*(0.077)*	*(0.045)*
45–54	−0.023	−0.058	−0.027
	*(0.057)*	*(0.075)*	*(0.046)*
Household income (£/€)			
25,000–44,999	0.066	0.164**	0.097**
	*(0.052)*	*(0.073)*	*(0.043)*
45,000–69,999	0.121**	0.178**	0.134***
	*(0.060)*	*(0.083)*	*(0.048)*
70,000–99,999	0.115	0.130	0.121**
	*(0.074)*	*(0.100)*	*(0.060)*
100,000	0.079	0.103	0.085
	*(0.086)*	*(0.129)*	*(0.072)*
No answer	−0.065	0.004	−0.044
	*(0.054)*	*(0.078)*	*(0.044)*
Level of education			
Secondary education	0.164	0.151	0.149*
	*(0.111)*	*(0.118)*	*(0.079)*
Vocational training	0.174	0.117	0.146*
	*(0.113)*	*(0.119)*	*(0.081)*
University degree	0.251**	0.104	0.194**
	*(0.111)*	*(0.120)*	*(0.080)*
Observations	**2140**	**1001**	**3141**
Adjusted R^2^	**0.012**	**0.011**	**0.042**
F-statistic	**2.352**	**1.605**	**8.009**
Prob > F	**0.001**	**0.048**	**0.000**
Baseline predicted score	**3.805**	**3.509**	**3.709**

The table shows results from OLS regressions where the dependent variable is the individual-level average response from five statements which measure ease of facilitation of the repair process (Q3|1, Q4|3,4, Q6|1,2), addressing RQ3. Columns 1 and 2 split the model into UK and Netherlands, respectively; column 3 reports results for the full sample including controls for country, gender, and country-gender effects (baseline category = UK male)

* *p* < 0.1, ** *p* < 0.05, *** *p* < 0.01. Robust standard errors in parentheses. Additional controls not shown for marital and employment status. Omitted baseline categories are for age: 55 +; household income: below 25,000; education: below secondary education

#### Ease of Facilitation

Our analysis documents gender differences in how consumers value ease of facilitation in the repair process, addressing Research Question 3. Women consistently demonstrate stronger preferences for streamlined, convenient repair procedures than men, although this pattern varies across countries. In the UK, ease of facilitation is a significantly more important factor for women than for men (Table 7, Column 1, *coeff.* 0.078, *p* <.05). This coefficient represents 1.95% of the 1–5 scale, or approximately 2% greater importance relative to the baseline predicted score of 3.805. In practical terms, if UK men, on average, rate ease of facilitation as moderately important (score of 3.8), UK women would rate it approximately 2% higher (3.88), indicating a stronger preference for streamlined processes.

An examination of the constituent items (Table 6) shows that this gender effect is driven primarily by UK women's greater concern about whether "the complaint procedure is easy and understandable" (4.08 for women vs 3.93 for men, *p* =.001) and their emphasis on sellers responding "quickly to complaints" (3.98 vs 3.89, *p* =.062). This pattern is consistent with the expectation articulated under RQ3 that time constraints and a preference for straightforward processes are particularly salient for women when deciding whether to pursue their right to choose repair.

However, this gender difference is not universal. There is no significant gender effect in the Netherlands (Table 7, Column 2). The interaction term in Column 3 (Female × Netherlands) is not statistically significant, indicating that the pattern of gender differences (a significant gap in the UK, no gap in the Netherlands) is not itself significantly different between the two countries. We find significant age and education effects, whereby ease of facilitation is more important for older and more educated consumers.

#### Costs

The largest country-by-gender differences in our data concern cost sensitivity in repair decisions, consistent with the expectation articulated under Research Question 4. Women show greater sensitivity to the financial aspects of the repair process than men; however, this gender effect is heavily influenced by national context. In the UK, costs are a significantly more important factor for women than for men (Table 8, Column 1, *coeff.* 0.072, *p* <.05). This 1.8% difference on the 1–5 scale represents approximately 1.8% greater importance relative to the baseline predicted score of 3.914. Practically, if UK men rate cost considerations as moderately important (score of 3.9), UK women would rate them as 3.97, a small but consistent increase in cost sensitivity that may influence their repair-related decisions.

**Table 8:** Regression analysis of factors influencing ‘Costs’

	(1) UK	(2) NL	(3) All
Female	0.072**	−0.061	0.072**
	*(0.036)*	*(0.056)*	*(0.036)*
Netherlands			−0.237***
			*(0.046)*
Female x Netherlands			−0.154**
			*(0.064)*
Age			
18–24	−0.160**	−0.091	−0.140**
	*(0.081)*	*(0.109)*	*(0.065)*
25–34	−0.266***	−0.126	−0.219***
	*(0.065)*	*(0.090)*	*(0.052)*
35–44	−0.202***	−0.005	−0.141***
	*(0.060)*	*(0.084)*	*(0.049)*
45–54	−0.169***	−0.133	−0.152***
	*(0.060)*	*(0.086)*	*(0.049)*
Household income (£/€)			
25,000–44,999	0.057	0.095	0.072
	*(0.054)*	*(0.087)*	*(0.046)*
45,000–69,999	0.049	0.087	0.061
	*(0.062)*	*(0.093)*	*(0.051)*
70,000–99,999	0.074	0.107	0.086
	*(0.076)*	*(0.118)*	*(0.064)*
100,000	0.066	0.146	0.088
	*(0.094)*	*(0.137)*	*(0.077)*
No answer	−0.077	−0.033	−0.062
	*(0.053)*	*(0.090)*	*(0.046)*
Level of education			
Secondary education	0.278***	0.104	0.195**
	*(0.107)*	*(0.138)*	*(0.084)*
Vocational training	0.362***	0.068	0.237***
	*(0.109)*	*(0.140)*	*(0.086)*
University degree	0.392***	0.203	0.303***
	*(0.107)*	*(0.141)*	*(0.086)*
Observations	**2140**	**1001**	**3141**
Adjusted R^2^	**0.019**	**0.004**	**0.049**
F-statistic	**3.459**	**1.386**	**8.788**
Prob > F	**0.000**	**0.124**	**0.000**
Baseline predicted score	**3.914**	**3.605**	**3.812**

The table shows results from OLS regressions where the dependent variable is the individual-level average response from four statements which measure costs of the repair process (Q3|2, Q4|1,2,6), addressing RQ4. Columns 1 and 2 split the model into UK and Netherlands, respectively; column 3 reports results for the full sample including controls for country, gender, and country-gender effects (baseline category = UK male)

* *p* < 0.1, ** *p* < 0.05, *** *p* < 0.01. Robust standard errors in parentheses. Additional controls not shown for marital and employment status. Omitted baseline categories are for age: 55 +; household income: below 25,000; education: below secondary education

This gender difference disappears in the Netherlands (Table 8, Column 2), where women and men display equivalent cost sensitivity. The negative and significant Female × Netherlands interaction in Column 3 (coeff. −0.154, p <.05) confirms that the gender gap in cost sensitivity differs across the two countries: the gap is 0.154 points larger in the UK than in the Netherlands, indicating that cost barriers more strongly affect UK women than their Dutch counterparts.

With regard to demographic variables, we find age and education effects in the UK: cost sensitivity is significantly lower among younger respondents and higher among those with more education.

#### Personal Attitudes

Our analysis documents gender differences in personal attitudes towards dispute resolution, with women exhibiting greater reluctance to engage in complaint processes than men, consistent with the expectation articulated under Research Question 5. However, as with cost sensitivity, these gender differences are moderated by national context. In the UK, women display significantly more negative personal attitudes towards dispute resolution than men (Table 9, Column 1). The coefficient for Female is positive and highly statistically significant (0.088, *p* <.01), representing a 2.2% difference on the 1–5 scale, or approximately 2.7% higher relative to the baseline predicted score of 3.213. This means that UK women report higher negative personal attitudes towards dispute resolution than UK men. If UK men rate the influence of negative attitudes as 3.2, UK women would rate them as 3.29, indicating greater reluctance to engage in complaint processes.

**Table 9:** Regression analysis of factors influencing ‘Personal Attitudes’

	(1) UK	(2) NL	(3) All
Female	0.088***	−0.019	0.088***
	*(0.030)*	*(0.043)*	*(0.030)*
Netherlands			−0.121***
			*(0.038)*
Female x Netherlands			−0.106**
			*(0.051)*
Age			
18–24	0.458***	0.342***	0.411***
	*(0.066)*	*(0.083)*	*(0.052)*
25–34	0.301***	0.333***	0.309***
	*(0.056)*	*(0.075)*	*(0.044)*
35–44	0.171***	0.189***	0.172***
	*(0.050)*	*(0.064)*	*(0.040)*
45–54	0.152***	0.158**	0.151***
	*(0.050)*	*(0.067)*	*(0.040)*
Household income (£/€)			
25,000–44,999	0.029	−0.084	0.006
	*(0.044)*	*(0.072)*	*(0.038)*
45,000–69,999	0.053	−0.111	0.009
	*(0.052)*	*(0.078)*	*(0.043)*
70,000–99,999	0.035	−0.035	0.021
	*(0.064)*	*(0.096)*	*(0.053)*
100,000	−0.008	0.048	0.019
	*(0.077)*	*(0.119)*	*(0.065)*
No answer	−0.078*	−0.099	−0.075*
	*(0.046)*	*(0.071)*	*(0.038)*
Level of education			
Secondary education	−0.140	0.093	−0.041
	*(0.097)*	*(0.099)*	*(0.069)*
Vocational training	−0.166*	0.121	−0.048
	*(0.100)*	*(0.102)*	*(0.072)*
University degree	−0.048	0.003	−0.003
	*(0.098)*	*(0.103)*	*(0.071)*
Observations	**2140**	**1001**	**3141**
Adjusted R^2^	**0.050**	**0.038**	**0.054**
F-statistic	**7.813**	**3.267**	**10.986**
Prob > F	**0.000**	**0.000**	**0.000**
Baseline predicted score	**3.213**	**3.039**	**3.157**

The table shows results from OLS regressions where the dependent variable is the individual-level average response from five statements which measure personal attitudes towards the repair process (Q3|3, Q4|5, Q6|3,4,5), addressing RQ5. Columns 1 and 2 split the model into UK and Netherlands, respectively; column 3 reports results for the full sample including controls for country, gender, and country-gender effects (baseline category = UK male)

* *p* < 0.1, ** *p* < 0.05, *** *p* < 0.01. Robust standard errors in parentheses. Additional controls not shown for marital and employment status. Omitted baseline categories are for age: 55 +; household income: below 25,000; education: below secondary education

This gender gap disappears in the Netherlands. In contrast to the UK pattern, there is no significant gender effect in the Netherlands (Table 9, Column 2, *coeff.* −0.019). The Female × Netherlands interaction term in Column 3 is negative and statistically significant (−0.106, *p* <.05). This interaction effect cancels out the positive gender effect observed in the UK for Dutch women: 0.088—0.106 = −0.018, which closely matches the non-significant −0.019 coefficient in the Netherlands-only model. This indicates that, while UK women show significantly more negative personal attitudes than UK men, the same gender gap does not exist in the Dutch context. Consistent with the expectation under RQ5, this gender pattern appears specifically in the UK, with national context moderating the relationship.

We observe significant age effects in both countries, with younger consumers exhibiting more negative personal attitudes than older ones. More educated consumers in the UK show lower levels of negative personal attitudes.

#### Preference for Alternative Solutions

Our analysis of gender differences in preferences for alternative solutions documents only limited patterns in relation to Research Question 6. Unlike the larger gender effects found in cost sensitivity and personal attitudes, preferences for alternatives to seller-provided repair show minimal gender variation. In the UK, there is no significant overall gender effect (Table 10, Column 1). A marginally significant effect is observed in the Netherlands (Table 10, Column 2, *coeff.* −0.095, *p* <.1), representing a 2.4% difference on the 1–5 scale, or approximately 3.6% lower relative to the baseline predicted score of 2.630. This suggests that Dutch women are slightly less inclined towards alternative options than men. If Dutch men rate their preference for alternatives as moderately high (score of 2.7), Dutch women would rate it as 2.6, suggesting a marginally stronger preference for seller-provided repair.

**Table 10:** Regression analysis of factors influencing ‘Preference for Alternative Solutions’

	(1) UK	(2) NL	(3) All
Female	−0.048	−0.095*	−0.048
	*(0.033)*	*(0.051)*	*(0.033)*
Netherlands			0.035
			*(0.043)*
Female x Netherlands			−0.043
			*(0.059)*
Age			
18–24	0.688***	0.647***	0.671***
	*(0.076)*	*(0.096)*	*(0.059)*
25–34	0.557***	0.581***	0.560***
	*(0.062)*	*(0.083)*	*(0.049)*
35–44	0.429***	0.357***	0.413***
	*(0.055)*	*(0.083)*	*(0.045)*
45–54	0.220***	0.253***	0.229***
	*(0.055)*	*(0.080)*	*(0.045)*
Household income (£/€)			
25,000–44,999	0.027	−0.066	0.005
	*(0.049)*	*(0.077)*	*(0.041)*
45,000–69,999	−0.065	−0.040	−0.049
	*(0.056)*	*(0.088)*	*(0.047)*
70,000–99,999	−0.051	−0.025	−0.039
	*(0.072)*	*(0.110)*	*(0.060)*
100,000	−0.077	−0.067	−0.069
	*(0.079)*	*(0.135)*	*(0.069)*
No answer	−0.044	0.024	−0.021
	*(0.047)*	*(0.078)*	*(0.040)*
Level of education			
Secondary education	−0.029	−0.204**	−0.109
	*(0.102)*	*(0.100)*	*(0.072)*
Vocational training	−0.077	−0.220**	−0.149**
	*(0.106)*	*(0.105)*	*(0.075)*
University degree	−0.032	−0.322***	−0.147**
	*(0.103)*	*(0.105)*	*(0.073)*
Observations	**2140**	**1001**	**3141**
Adjusted R^2^	**0.123**	**0.098**	**0.116**
F-statistic	**17.894**	**7.117**	**21.410**
Prob > F	**0.000**	**0.000**	**0.000**
Baseline predicted score	**2.622**	**2.630**	**2.628**

The table shows results from OLS regressions where the dependent variable is the individual-level average response from four statements which measure preference for alternative solutions (Q3|4,5,6, Q6|6), addressing RQ6. Columns 1 and 2 split the model into UK and Netherlands, respectively; column 3 reports results for the full sample including controls for country, gender, and country-gender effects (baseline category = UK male)

* *p* < 0.1, ** *p* < 0.05, *** *p* < 0.01. Robust standard errors in parentheses. Additional controls not shown for marital and employment status. Omitted baseline categories are for age: 55 +; household income: below 25,000; education: below secondary education

However, the Female × Netherlands interaction term is not significant, meaning we cannot statistically conclude that gender effects differ between countries, though the descriptive pattern suggests some variation. Overall, the gender patterns in relation to RQ6 are limited and country-specific. The most consistent predictors of preferences for alternative resolution methods are age and education rather than gender: younger consumers in both countries are significantly more inclined towards alternative solutions, while those with higher education are less so.

Taken together, the effect sizes reported in Table 6 confirm that the gender differences across all four factors are modest. Even where statistically significant, Cohen's *d* remains below 0.2 for almost every comparison, and the largest single effect (Q3, item 6 in the Netherlands) reaches only *d* = 0.29. These small effect sizes are consistent with the regression coefficients reported above and reinforce our characterisation of the gender differences as systematic in direction but limited in magnitude. They underscore that the central contribution of this study lies in the consistency of these patterns across questions and their alignment with the legal framework, rather than in the size of any individual difference.

#### What Predicts Willingness to Enforce?

Finally, we move beyond assessing gender differences in individual barriers to address a broader question: what are the strongest overall predictors of a consumer's willingness to enforce their rights? For this analysis, we focus on the full pooled sample (Column 3), in order to maximize statistical power. Our outcome variable is based on responses to the survey item "Not applicable, it is unlikely I would stop pursuing my complaint", which represents a commitment to enforcement. We then use our four composite factors as predictors in a regression model, with results presented in Table 11.

**Table 11:** Regression analysis of predictors of consumers' Willingness to Enforce their Rights

	(1) UK	(2) NL	(3) All
Female	−0.040**	−0.014	−0.039**
	*(0.019)*	*(0.026)*	*(0.019)*
Netherlands			−0.077***
			*(0.023)*
Female x Netherlands			0.025
			*(0.031)*
Ease of Facilitation	0.224***	0.216***	0.220***
	*(0.020)*	*(0.022)*	*(0.015)*
Costs	−0.108***	−0.077***	−0.095***
	*(0.016)*	*(0.017)*	*(0.012)*
Personal Attitudes	0.090***	0.060**	0.078***
	*(0.018)*	*(0.024)*	*(0.015)*
Preference for Alternative Solutions	−0.175***	−0.194***	−0.183***
	*(0.013)*	*(0.018)*	*(0.010)*
Age			
18–24	−0.140***	−0.054	−0.102***
	*(0.047)*	*(0.054)*	*(0.035)*
25–34	0.037	−0.010	0.023
	*(0.036)*	*(0.046)*	*(0.028)*
35–44	−0.042	0.016	−0.026
	*(0.031)*	*(0.042)*	*(0.025)*
45–54	0.063*	0.055	0.061**
	*(0.033)*	*(0.043)*	*(0.026)*
Household income (£/€)			
25,000–44,999	−0.014	−0.039	−0.023
	*(0.028)*	*(0.041)*	*(0.023)*
45,000–69,999	−0.001	0.008	0.000
	*(0.032)*	*(0.045)*	*(0.026)*
70,000–99,999	0.093**	−0.030	0.054
	*(0.042)*	*(0.054)*	*(0.033)*
100,000	−0.026	0.062	−0.001
	*(0.049)*	*(0.070)*	*(0.040)*
No answer	0.108***	0.033	0.084***
	*(0.028)*	*(0.043)*	*(0.024)*
Level of education			
Secondary education	0.112**	0.016	0.058
	*(0.056)*	*(0.056)*	*(0.040)*
Vocational training	0.136**	−0.023	0.062
	*(0.058)*	*(0.058)*	*(0.041)*
University degree	0.080	0.003	0.034
	*(0.056)*	*(0.057)*	*(0.040)*
Observations	**2140**	**1001**	**3141**
Adjusted R^2^	**0.208**	**0.207**	**0.217**
F-statistic	**31.910**	**14.130**	**46.362**
Prob > F	**0.000**	**0.000**	**0.000**
Baseline predicted score	**0.364**	**0.301**	**0.343**

The table shows results from OLS regressions where the dependent variable is a binary measure of the consumers' willingness to enforce their rights (1 = unlikely to stop pursuing a complaint, 0 = otherwise). The independent variables are the four summary factors and demographic controls. Columns 1 and 2 split the model into UK and Netherlands, respectively; column 3 reports results for the full sample including controls for country, gender, and country-gender effects (baseline category = UK male)

* *p* < 0.1, ** *p* < 0.05, *** *p* < 0.01. Robust standard errors in parentheses. Additional controls not shown for marital and employment status. Omitted baseline categories are for age: 55 +; household income: below 25,000; education: below secondary education

The results show that all four factors are significant predictors of a consumer's willingness to enforce their rights. Comparing the magnitude of the coefficients in the full model (Column 3) shows the relative importance of these factors. Ease of facilitation (*coeff.* 0.220, *p* <.001) is the largest positive predictor, while preference for alternative solutions (*coeff.* −0.183, *p* <.001) is the largest negative predictor. To interpret these effects: a one-point increase in ease of facilitation (e.g., moving from "somewhat important" to "very important" on the 1–5 scale) increases the probability of persistent enforcement by 22 percentage points. Conversely, a one-point increase in preference for alternatives decreases enforcement probability by 18.3 percentage points. These results suggest that, across consumers in both countries, the largest driver of enforcement is a process that is simple, timely, and convenient, whereas the largest barrier is a pre-existing preference for alternatives to a repair claim. Costs (*coeff.* −0.095) and personal attitudes (*coeff.* 0.078) are also statistically significant, with smaller effects on the decision to enforce. They represent 9.5 and 7.8 percentage point changes, respectively. The four factors together explain 21.7% of the variance in willingness to enforce (Column 3), with ease of facilitation and preference for alternatives showing the largest coefficients.

In relation to our gender focus, the results also show an overall gender effect in enforcement willingness. Women are 3.9 percentage points less likely than men to enforce their rights, representing an 11% decrease relative to the baseline predicted enforcement rate of 0.343 (34.3%). This gender enforcement gap is primarily driven by the UK context, where women display significantly lower enforcement willingness than men (Table 11, Column 1, *p* <.05). In contrast, no significant gender difference is observed in the Netherlands (Table 11, Column 2, *p* >.1). Since the Female × Netherlands interaction term is not statistically significant, we cannot conclude that gender effects differ between the two countries.

### Summary of Research Questions and Result

To facilitate interpretation and enable comparison across our six sub-questions, Table 12 presents effect sizes as percentage changes relative to baseline predicted values, rather than raw coefficients. This approach allows for meaningful comparisons across variables measured on different scales (e.g., 0–2 adherence to legal right index versus 1–5 factor scales) and provides a more intuitive understanding of practical significance. While detailed regression coefficients remain available in Tables 4, 5, and 7, 8, 9, 10 the summary below prioritises accessibility and cross-RQ comparison to support policy interpretation.

**Table 12:** Summary of research questions and results

	UK			Netherlands		
Research Question	Sup	Effect	Source	Sup	Effect	Source
RQ1 Adherence to the legal right to choose repair	✓	7% lower on the legal right index (p = 0.042); 10% less likely to select the seller (p = 0.006)	Table 3, Q1 + Q2	~	6% lower on the legal right index (p = 0.063); 8% less likely to select the seller (p = 0.036)	Table 3, Q1 + Q2
RQ2 Adherence to procedural conditions	✓	7% higher on the procedural conditions index (p = 0.008)	Table 3, Q5	✓	14% higher on the procedural conditions index (p = 0.009)	Table 3, Q5
RQ3 Ease of facilitation more important for women	✓	2% higher importance (p = 0.026*)	Table 7, Col 1	✗	1% higher (p = 0.407*)	Table 7, Col 2
RQ4 Costs more important for women (stronger in UK)	✓	1.8% higher importance (p = 0.046*)	Table 8, Col 1	✗	2% lower (p = 0.276*)	Table 8, Col 2
RQ5 Personal attitudes a greater barrier for women	✓	2.7% higher influence of negative attitudes (p = 0.003*)	Table 9, Col 1	✗	No effect (p = 0.660*)	Table 9, Col 2
RQ6 Preference for alternative solutions	✗	2% lower preference (p = 0.147*)	Table 10, Col 1	~	4% lower preference (p = 0.063*)	Table 10, Col 2

p-values marked with asterisk (*) were calculated from reported coefficients and standard errors using t = coefficient/standard error, then converted to p-value assuming normal distribution. p-values without asterisk are from original statistical output in Table 3. Regression results control for age, education, and income. Effect sizes for RQ1-RQ2 represent percentage point differences in mean scores. Effect sizes for RQ3-RQ6 represent percentage differences relative to baseline predicted values from regression models

Key: For RQ3, RQ4 and RQ5 (directional), ✓ = pattern consistent with the directional expectation; ~ = marginal pattern; ✗ = no significant gender difference. For RQ1, RQ2 and RQ6 (exploratory), ✓ = gender difference observed at conventional significance; ~ = marginal pattern; ✗ = no significant gender difference. Effect strength is based on regression coefficients and t-tests

- RQ1 – Adherence to the Legal Right: Gender differences are consistently observed, although they vary in statistical significance across countries. In the UK, women score 7% lower than men (*p* =.042) and are 10% less likely to select the seller, who bears the repair obligation under the CSD (*p* =.006). In the Netherlands, the gap as to whom to approach with a repair request persists (8% fewer women, *p* =.036), although overall differences in adherence to the legal right are not statistically significant (*p* =.063).
- RQ2 – Adherence to Procedural Conditions: Women's responses are more often consistent with legal entitlements than men's across both countries. In the UK, women score 7% higher than men (*p* =.008), and in the Netherlands, 14% higher (*p* =.009).
- RQ3 – Ease of Facilitation: In the UK, women rate facilitation as 2% more important than men (*p* =.026), whereas no gender difference is observed in the Netherlands (*p* =.407). The cross-national variation suggests that context may influence the salience of operational convenience for women.
- RQ4 – Costs: Women show greater sensitivity to financial aspects of the repair process. In the UK, women show 1.8% higher cost sensitivity than men (*p* =.046), while in the Netherlands, women show 2% lower cost sensitivity (*p* =.276). The statistically significant interaction effect indicates that gender differences in cost sensitivity vary by country.
- RQ5 – Personal Attitudes: In the UK, women report 2.7% more negative personal attitudes towards dispute resolution than men (*p* =.003), whereas no significant gender difference is found in the Netherlands (*p* =.660). The significant interaction effect suggests that national context can moderate or eliminate gender differences in this domain.
- RQ6 – Alternative Solutions: Gender differences in preference for alternatives to seller-provided repair are minimal. In the UK, women show 2% lower preference for alternatives (*p* =.147, non-significant), while in the Netherlands, women show 4% lower preference (*p* =.063, marginally significant). The absence of a significant interaction effect suggests that gender differences in this domain are small and consistent across countries.

Taken together, the results document a nuanced pattern of gender effects that varies across dimensions of consumer behaviour and between national contexts. These findings raise important questions for the design and implementation of consumer protection frameworks, which we address in the following discussion.

## Discussion

### Gendered Patterns in Consumer Rights: Core Findings

Our results document gender differences in adherence to consumer rights and enforcement behaviour, with variation across national contexts. Overall, women face distinct barriers to enforcing their consumer rights. Women score lower than men on the basic literacy of and adherence to the legal right, particularly in the UK context. However, they show higher adherence to procedural conditions, more often selecting responses consistent with the practical requirements for exercising the right to choose repair. This suggests that gender shapes adherence to consumer contract law in ways that interact with both substantive and procedural dimensions.

Enforcement barriers also differ by gender. In the UK, women express greater concern than men about procedural complexity, financial costs, and conflict-related aspects of pursuing repair. These differences are smaller in the Netherlands.

Despite these variations in adherence and specific barriers, a consistent pattern is observed: women are less likely than men to enforce their right to choose repair. This suggests that the cumulative effect of gendered obstacles may undermine effective rights enforcement for women. Our research has identified a number of systemic barriers that policy makers could use as guidance for addressing.

### Interpreting the Gendered Pattern

Our research questions were motivated by a candidate mechanism drawn from psychology and economics of gender differences in behaviour, developed above: conflict and competition avoidance (Holt & DeVore, 2005; Niederle & Vesterlund, 2007), time constraints (Craig & Mullan, 2011), complaint-related guilt (Kayal et al., 2017), and gendered differences in information processing and consumer experience (Krizan et al., 2022; Meyers-Levy & Loken, 2015). With the results in hand, we can ask which of these the data favour. The organising puzzle is not the enforcement gap alone but the divergence within legal literacy itself: women's responses are less often aligned with the substantive right yet more often aligned with the procedural conditions for exercising it. A satisfactory account has to explain that specific asymmetry, not merely a general gender difference.

Most of the mechanisms we invoked bear on enforcement rather than on this split. The conflict-avoidance, complaint-related guilt, and time-constraint channels motivating RQ3 and RQ5 plausibly depress willingness to enforce and raise the salience of cost and procedural barriers, but they are largely silent on why women's recognition of the procedural position should exceed men's while their grasp of the substantive entitlement falls short. The one account that predicts the asymmetry directly is the differential-experience channel behind RQ1, that is, the idea that routine and repeated dealings with sellers build practical, situation-specific familiarity even where formal legal knowledge does not follow (Krizan et al., 2022; Meyers-Levy & Loken, 2015). As the primary managers of most household purchases (Krizan et al., 2022), women accumulate practical familiarity with after-sales encounters that sharpens recognition of improper seller demands without necessarily conveying the more abstract doctrinal entitlement of who owes the obligation and what may be chosen. On the present evidence this is the most economical explanation of the split, with the enforcement-side mechanisms operating downstream of it and largely independently. The information-processing account also raised under RQ1 is consistent with this reading but is not something our data can speak to, since we measure no processing style.

Two features of the results support this interpretation. First, the substantive-procedural divergence holds across both countries, which is what an experience-based account predicts and what a purely institutional one would not. Second, the barrier-related gaps in cost sensitivity and personal attitudes appear in the UK but not the Netherlands; that contextual variation weighs against treating any of these channels as a fixed feature of gender and points instead to socialisation and institutional setting. We cannot go further with cross-sectional data that contain no direct measure of experience, processing or motivation, so we carry forward differential experience as the leading candidate and the enforcement-side mechanisms as complementary. Cleanly disentangling these mechanisms from one another would require data designed for that purpose, such as experimental or longitudinal studies able to isolate each one. This is the question we believe most warrants the further investigation that our policy proposals, set out below, presuppose.

### Why Some Gender Effects Vary: The Role of Context

While the overall pattern of gendered enforcement gaps is consistent, our findings also show that the type and intensity of these effects vary across national contexts. This is particularly evident in relation to psychological barriers to enforcement. The expectation under RQ5 that women would be more discouraged by negative personal attitudes towards dispute resolution is borne out in the UK, where women report greater reluctance to engage in complaint processes, lower expectations of successful outcomes, and heightened emotional discomfort in pursuing claims. By contrast, in the Netherlands, these gender differences disappear, suggesting that institutional or cultural factors may moderate these effects.

Not all gender differences anticipated in the literature were borne out. The patterns in relation to RQ6 are limited, with a marginal effect only in the Netherlands. This suggests that, although women may hold stronger environmental values in general, these do not necessarily translate into stronger preferences for pursuing the repair of defective goods when practical considerations such as cost or convenience are involved.

These contrasting findings highlight the context-dependence of gender effects. Some aspects of gendered behaviour, such as personal attitudes towards dispute resolution, may vary across national contexts, while others, such as environmental motivation, may show greater consistency across countries. This pattern underscores the need to view gender not as a fixed determinant, but as a dynamic factor shaped by contextual forces. The precise mechanisms, whether institutional, cultural, or otherwise, that drive these variations require further investigation.

### Methodological Limitations of Cross-National Comparisons

While our cross-national findings suggest that institutional frameworks may influence how gender affects consumer rights enforcement, several alternative explanations are plausible. The observed differences between the UK and the Netherlands could reflect more than institutional design alone, and caution is therefore warranted in making causal inferences based on cross-sectional comparative data.

First, the difference in sample sizes between the two countries (UK: 2,140; Netherlands: 1,001) affects the statistical power to detect effects. The absence of statistically significant gender effects in the Netherlands may reflect insufficient power to detect small but meaningful differences, rather than a genuine absence of gender disparities. This is an important consideration when interpreting null results, as lack of significance does not necessarily imply that no difference exists.

Second, cultural differences beyond formal institutional arrangements may contribute to the observed variation. The Netherlands and the UK differ in broader societal norms, such as those concerning gender equality, workplace policies, social norms around conflict and assertiveness, and attitudes towards consumer rights (European Institute for Gender Equality, 2023). Cross-national research documents cultural variation in workplace gender equality, with the Netherlands exhibiting markedly different part-time work arrangements and gender role distributions compared to the UK (OECD, 2019). Cultural factors also shape consumer complaint behaviours and service expectations, with consumers in different national contexts displaying varying propensities to voice dissatisfaction and seek redress (Ngai et al., 2007; Schuckert et al., 2015). Cross-cultural analysis spanning 76 countries shows that values emphasizing tolerance and self-expression have diverged across national cultures, including within Europe (Jackson & Medvedev, 2024). Such unmeasured cultural influences could shape how gender affects consumer behaviour, independently of formal consumer protection frameworks.

Third, while our models control for a range of demographic and economic factors, these may not fully capture the contextual variation influencing gendered behaviour. Structural differences in labour markets, family policies, or levels of economic inequality could moderate the relationship between gender and consumer behaviour in ways our study cannot fully isolate.

Fourth, the implementation of consumer protection policies, both in timing and in practice, may be as important as their formal design. Similar legal frameworks can operate very differently depending on enforcement priorities, resource allocation, or administrative culture, all of which lie beyond the scope of our study.

Taken together, these considerations suggest that, while institutional design remains a plausible explanation for cross-national differences in gender effects, definitive causal claims require stronger empirical foundations. Future research employing longitudinal designs, policy variation studies, or natural experiments would provide more robust insights into how institutional factors shape gendered patterns in consumer behaviour.

### Advancing Understanding of Gendered Consumer Behaviour

Our study contributes to a largely underexplored area in consumer protection research: how gender shapes consumer legal literacy and enforcement behaviour. While previous research has documented gender differences in purchasing patterns and complaint behaviour, this study is among the first to empirically examine how gender influences adherence to and enforcement of specific consumer rights. By applying a gender lens, we uncover patterns that would remain hidden if consumers were treated as a homogeneous group.

Our methodological innovations enable a more nuanced analysis of gendered consumer behaviour. First, we moved beyond self-reported awareness of legal rights by employing scenario-based measures of how consumers respond to specific repair situations, capturing adherence rather than self-reported knowledge. This shows gendered patterns in when women and men act consistently with their legal rights. Second, examining these patterns further, we developed novel indices capturing adherence to both the substantive legal right and the procedural conditions for exercising it, showing that women's responses are more often consistent with procedural conditions, while men's are more often consistent with the substantive legal right. Third, our cross-national design indicates that gender effects are not fixed but may vary according to institutional design.

Overall, our findings underline the value of applying a gender lens to consumer protection research. Our multivariate regression analyses, incorporating interaction terms between gender and country, show how national frameworks can either exacerbate or reduce gender disparities.

### Practical Significance of Gender Differences

Although the gender differences we document are modest in magnitude, as the effect sizes reported in Table 6 confirm, they carry meaningful real-world implications for consumer protection policy. In the UK, women's greater sensitivity to repair-related costs poses a barrier to enforcement. Women consistently report that cost-related concerns influence their repair decisions, suggesting that even small increases in perceived costs can deter enforcement. Policy interventions that emphasize the free nature of the right to choose repair could therefore particularly benefit women.

Larger gender differences appear in adherence to procedural conditions. On average, women's responses are consistent with 0.2 to 0.3 more procedural conditions than men's, placing them in a stronger position to recognise when sellers are improperly demanding payment for repair services that should be free under consumer protection law. Despite this, women remain less likely to pursue claims, highlighting that recognising the procedural position alone is insufficient. Removing practical barriers such as cost and complexity is essential to achieving gender-equitable access to consumer protection. A robustness check decomposing the index (Tables 13 and 14) locates this advantage in the "pay X" items rather than the "prove X" items, consistent with our reading that women are more likely to reject attempts to shift repair costs onto them, which is the legally correct position.

**Table 13:** Robustness check: adherence to procedural conditions index excluding “Pay for X” Items

	(1) UK	(2) NL	(3) All
Female	0.015	0.067	0.022
	*(0.029)*	*(0.056)*	*(0.029)*
Netherlands			−0.147***
			*(0.045)*
Female x Netherlands			0.025
			*(0.062)*
Age			
18–24	−0.072	−0.117	−0.125**
	*(0.067)*	*(0.110)*	*(0.060)*
25–34	−0.053	−0.153*	−0.097**
	*(0.053)*	*(0.093)*	*(0.047)*
35–44	−0.087*	0.001	−0.066
	*(0.049)*	*(0.094)*	*(0.045)*
45–54	−0.098*	−0.027	−0.079*
	*(0.051)*	*(0.089)*	*(0.045)*
Household income (£/€)			
25,000–44,999	0.065	0.177**	0.091**
	*(0.044)*	*(0.086)*	*(0.040)*
45,000–69,999	0.087*	0.179*	0.110**
	*(0.051)*	*(0.094)*	*(0.046)*
70,000–99,999	0.171***	0.196*	0.169***
	*(0.065)*	*(0.118)*	*(0.058)*
100,000	0.087	0.394**	0.173**
	*(0.078)*	*(0.169)*	*(0.076)*
No answer	−0.034	0.187**	0.030
	*(0.043)*	*(0.090)*	*(0.040)*
Level of education			
Secondary education	−0.032	0.084	0.037
	*(0.080)*	*(0.126)*	*(0.072)*
Vocational training	−0.018	0.082	0.041
	*(0.083)*	*(0.128)*	*(0.074)*
University degree	−0.014	0.338***	0.125*
	*(0.082)*	*(0.130)*	*(0.074)*
Observations	**2140**	**1001**	**3141**
Adjusted R^2^	**0.012**	**0.037**	**0.018**
F-statistic	**2.387**	**2.713**	**3.874**
Prob > F	**0.001**	**0.000**	**0.000**
Baseline predicted score	**0.784**	**0.667**	**0.736**

The table shows results from OLS regressions where the dependent variable is the adherence to procedural conditions index restricted to the three “prove X” items of Q5 (items 1–3, range 0–3). The six Q5 statements combine demands that the consumer prove something (items 1–3) with demands that the consumer pay something (items 4–6), which may tap distinct constructs; the three “pay for X” items have therefore been dropped here and are examined separately in Table 14 (range 0–3). Columns 1 and 2 split the model into UK and Netherlands, respectively; column 3 reports results for the full sample including controls for country, gender, and country-gender effects (baseline category = UK male)

^*^ *p* < 0.1, ** *p* < 0.05, *** *p* < 0.01. Robust standard errors in parentheses. Additional controls not shown for marital and employment status. Omitted baseline categories are for age: 55 +; household income: below 25,000; education: below secondary education

**Table 14:** Robustness check: adherence to procedural conditions index excluding “Prove X” items

	(1) UK	(2) NL	(3) All
Female	0.172***	0.274***	0.168***
	*(0.050)*	*(0.080)*	*(0.050)*
Netherlands			−0.274***
			*(0.067)*
Female x Netherlands			0.081
			*(0.091)*
Age			
18–24	−1.170***	−0.648***	−0.981***
	*(0.124)*	*(0.147)*	*(0.093)*
25–34	−0.770***	−0.241*	−0.579***
	*(0.095)*	*(0.133)*	*(0.077)*
35–44	−0.674***	−0.222*	−0.539***
	*(0.083)*	*(0.130)*	*(0.070)*
45–54	−0.315***	−0.302**	−0.304***
	*(0.084)*	*(0.125)*	*(0.069)*
Household income (£/€)			
25,000–44,999	0.005	0.367***	0.089
	*(0.076)*	*(0.120)*	*(0.064)*
45,000–69,999	0.059	0.406***	0.137*
	*(0.089)*	*(0.130)*	*(0.073)*
70,000–99,999	0.322***	0.290*	0.283***
	*(0.108)*	*(0.171)*	*(0.092)*
100,000	0.041	0.285	0.096
	*(0.132)*	*(0.209)*	*(0.113)*
No answer	−0.078	0.096	−0.046
	*(0.075)*	*(0.121)*	*(0.064)*
Level of education			
Secondary education	0.075	0.243	0.148
	*(0.147)*	*(0.184)*	*(0.115)*
Vocational training	0.147	0.193	0.189
	*(0.152)*	*(0.189)*	*(0.119)*
University degree	0.111	0.357*	0.210*
	*(0.150)*	*(0.188)*	*(0.117)*
Observations	**2140**	**1001**	**3141**
Adjusted R^2^	**0.104**	**0.060**	**0.092**
F-statistic	**14.626**	**5.171**	**17.761**
Prob > F	**0.000**	**0.000**	**0.000**
Baseline predicted score	**2.032**	**1.805**	**1.957**

The table shows results from OLS regressions where the dependent variable is the adherence to procedural conditions index restricted to the three “pay for X” items of Q5 (items 4–6, range 0–3): paying for delivery to the seller, paying for repair itself, and paying for return shipping. Items 1–3 (“prove X”) have been dropped and are examined separately in Table 13. Together, the two decompositions map where the gender pattern in the procedural-conditions index lives: the gender gap is concentrated in items 4–6 and is absent from items 1–3, consistent with our framing of Q5 as measuring adherence to procedural conditions rather than abstract legal knowledge. Columns 1 and 2 split the model into UK and Netherlands, respectively; column 3 reports results for the full sample including controls for country, gender, and country-gender effects (baseline category = UK male)

^*^ *p* < 0.1, ** *p* < 0.05, *** *p* < 0.01. Robust standard errors in parentheses. Additional controls not shown for marital and employment status. Omitted baseline categories are for age: 55 +; household income: below 25,000; education: below secondary education

Institutional context appears to make a difference, suggesting the practical significance of institutional design choices. The cost sensitivity gap between UK men and women disappears in the Dutch context, as does the influence of differences in attitudes towards dispute resolution. This contextual variation suggests that gender disparities in enforcement are not inevitable or biologically predetermined, and that they may be addressed through appropriate policy interventions. For policymakers, this points to gender equity in consumer protection as an achievable goal through careful legal design.

### Policy Implications for Gender-Equitable Consumer Protection

Our findings indicate that current consumer protection models may inadvertently create unequal access to rights along gendered lines. Targeted policy measures may help to address this. We suggest several directions for policy aimed at both removing barriers and building on existing strengths.

First, while women's responses are consistent with procedural conditions of the repair process, they are less likely to select the seller as the appropriate first point of contact when a product malfunctions. Policymakers could address this by developing communication strategies that meet these information needs, for example, ensuring sellers' contact details and responsibilities are more prominently displayed. Such initiatives could involve tailoring information campaigns to build on women's existing adherence to procedural conditions while addressing remaining gaps.

Second, women's greater cost sensitivity in the UK (absent in the Dutch context) points to actionable levers. Emphasizing the cost-free nature of the right to choose repair, mandating clearer disclosures from sellers, and strengthening enforcement against unwarranted charges may help reduce gendered enforcement gaps. The absence of this gap in the Netherlands warrants further research into how the right to choose repair is communicated across contexts, and what factors might explain these cross-national differences, as this could identify approaches to reducing gender-based barriers that are transferable to other jurisdictions.

Third, streamlining procedures may improve practical access to remedies for women. As feminist legal scholarship has long argued, legal systems tend to reflect and reinforce existing power structures (Smart, 1977). Empirical research on employment discrimination litigation similarly shows that legal rights do not automatically translate into effective enforcement, with barriers affecting different groups' ability to pursue claims (Nielson & Nelson, 2005). Addressing such barriers is important to turning legal rights into lived realities, supporting consumer protection that is inclusive across genders. Potential policy interventions include administrative simplification, accessible repair request channels, and shorter resolution timelines.

A note of caution is warranted about the kind of intervention these findings support. An objection could be raised to tailoring consumer protection to behavioural tendencies more commonly observed among women, such as greater conflict avoidance or risk aversion, on the grounds that this may risk entrenching those tendencies rather than expanding consumers' capacity to act. This concern is analogous to clinical contexts, where short-term "safety behaviours" that reduce immediate discomfort may, over time, reinforce patterns of avoidance (Salkovskis et al., 1999). We take this concern seriously, however, it does not undermine the measures proposed here for two reasons. First, our recommendations target structural barriers rather than individual dispositions: clearer disclosure of the cost-free nature of the right, more prominent information about the seller's responsibilities, enforcement against improper charges, and simpler procedures remove obstacles that the law's own design has allowed to persist. They lower the cost of acting. They do not lower the standard of what consumers are expected to do, nor do they substitute for consumers' agency. Second, the law is never behaviourally neutral. The existing framework already shapes how consumers engage with their rights and our findings suggest it may do so around a male-default baseline that is inherited rather than chosen. The relevant question is therefore not whether the law should influence behaviour, but whether it does so deliberately and inclusively or by default. Understood this way, reducing systemic barriers and supporting long-term empowerment are complementary rather than opposed: a consumer who is not deterred at the outset by avoidable cost and complexity is better placed, not worse, to exercise agency in asserting the right.

The cross-national variation in our findings suggests that gender inequalities in consumer protection are not inevitable. While our study cannot definitively isolate the reasons for these differences, further investigation into the specific institutional, cultural, or communicative factors that promote more equitable outcomes could inform approaches to reducing gender-based barriers. Such research could establish best practices for other jurisdictions and support EU-wide policy harmonisation efforts.

### Directions for Future Research

Our study also opens several avenues for future research that could deepen the understanding of gender dynamics in consumer protection and extend our findings to broader contexts. The gender patterns observed raise important questions about the interaction between legal frameworks and social identities, and about which policy approaches are most effective at promoting equitable access to legal rights.

First, broadening the scope of products studied would be valuable. Our focus on dishwashers ensured product neutrality but may have excluded dynamics more salient with other consumer goods. Products with stronger gender associations, different price points, or varying levels of complexity might show different patterns of adherence and enforcement behaviour. Future research should explore whether our findings generalise across product categories or whether product-specific policies are needed.

Second, the cross-national variation we observe requires deeper investigation. While our results suggest that national contexts matter, we do not yet know which aspects of consumer protection frameworks, legal design, enforcement mechanisms, cultural norms, or institutional support, are most influential. Clarifying this would enhance the precision of policy development.

Third, longitudinal studies would shed light on the durability of gendered patterns. Our cross-sectional design cannot capture how gender effects evolve over time as repair rights frameworks develop and consumer awareness grows. Longitudinal research would help determine whether gender gaps persist, diminish, or change character as consumer protection regimes mature and as broader gender equality initiatives take effect. This would also help policymakers understand whether gender-sensitive interventions require ongoing attention or represent transitional measures.

Finally, future research should investigate the underlying mechanisms driving the gender differences we identify. While our study documents these disparities, it does not determine whether they stem from socialisation processes, resource constraints, time scarcity, differences in information processing, or other factors. Understanding these drivers would help shift policy responses from managing symptoms to tackling root causes of inequality in consumer rights enforcement. This research could not only strengthen consumer protection but also contribute to a broader understanding of how legal frameworks can promote substantive equality across diverse groups.

## Conclusion

This study documents gender differences in how consumers engage with and enforce their right to choose repair, raising questions about whether gender-neutral legal frameworks inevitably produce equitable outcomes. By examining attitudes and obstacles to enforcement of the right to choose repair across two European countries, we show that women face distinct barriers, including greater sensitivity to financial costs, procedural complexity, and conflict-related concerns, even as their responses are more often consistent with the procedural conditions for exercising the right. This pattern means that those who recognise the procedural position may be less likely to enforce their rights.

Our findings raise questions about the "average consumer" benchmark that dominates European consumer law, which may disadvantage women and create gendered enforcement gaps that undermine the purpose of consumer protection. In the UK, women are 11% less likely than men to pursue their right to choose repair, show 1.8% higher cost sensitivity, and 2.7% greater reluctance towards dispute resolution. While these differences may appear modest in statistical terms, they translate into meaningful real-world barriers that can prevent women from accessing the protection to which they are legally entitled.

The contrast with the Netherlands, where many gender gaps are not statistically significant, suggests that gender inequalities in consumer protection are not inevitable. Policy design, institutional frameworks, and cultural norms likely influence gender equity, though further research is needed to identify specific causal mechanisms. The Dutch case suggests that more gender-neutral access to consumer rights is achievable, whether through policy design, cultural factors, institutional support, or other unmeasured influences.

These insights extend beyond the right to choose repair, raising broader questions about how ostensibly neutral legal frameworks may perpetuate substantive gender inequality. Our evidence suggests that the "average consumer" benchmark may privilege certain patterns of legal engagement while disadvantaging others, undermining consumer law's goal of levelling the playing field between consumers and businesses.

The implications for sustainable consumption are particularly significant. As European policy increasingly promotes repair over replacement to advance circular economy goals, basing this transition on consumer protection frameworks that exclude certain groups from effective participation risks entrenching inequality. If the right to choose repair is to support sustainable consumption, it must be genuinely accessible to all consumers, not only to those whose knowledge, financial resources, and tolerance for conflict align with existing frameworks.

Our research points towards a re-orientation of consumer protection towards substantive gender equality. Women's higher adherence to the procedural conditions of their right to choose repair is a valuable but under-utilised asset within current frameworks. Complaint processes could address women's greater sensitivity to costs and procedural complexity, rather than framing these as individual shortcomings. Success could be measured not solely by the rights guaranteed in legislation, but by their equitable enforcement across all segments of the consumer population.

The path forward involves explicit attention to gender in the design and implementation of consumer protection measures, recognising that gender-blind approaches can yield biased outcomes. Policy interventions might include emphasizing the cost-free nature of the right to choose repair to address women's cost sensitivity, streamlining procedures to reduce enforcement barriers, and creating accessible claim channels that accommodate different levels of comfort with confrontational processes.

Our findings suggest that the promise of consumer law to protect all consumers equally is unlikely to be fulfilled while gendered barriers to enforcement persist. Effective consumer protection benefits from legal frameworks informed by an explicit understanding of diverse consumer experiences, supporting an inclusive transition to sustainable consumption.

## Data Availability

The questionnaire, statistical code, and replication documentation are available from the authors upon request. The underlying dataset may be made available upon reasonable request, subject to YouGov's permission. The survey microdata are subject to YouGov's intellectual property rights and terms of use (Source: YouGov Plc 2023 © All rights reserved) as they were collected by YouGov Plc from its online panels in the United Kingdom and the Netherlands under a commercial research services agreement.

## Appendix

To make our results accessible to a broad audience, this section explains our statistical methods in non-technical terms. We employ three main tools to analyse the data and include a section on how to interpret the results produced by these analytical tools.

### Simple Comparisons

When comparing the average responses between two groups – for instance, men and women – we use a simple *t*-test. This test helps us assess whether the difference observed in our sample is likely to reflect a genuine difference in the wider population or whether it may have arisen by chance. The key result is the *p*-value, which represents the probability of observing such a difference if, in reality, no difference exists. If the *p*-value is small (by convention, less than.05, or 5%), the result is considered statistically significant, meaning it is unlikely to be due to random variation alone. For example, in Table 3, we observe that for the adherence to the legal right index in the UK, men scored an average of 1.03 and women scored 0.96. The *p*-value for this difference is.042. Because.042 is less than.05, we can conclude that this difference is statistically significant.

### Regression Analysis: Understanding Complex Relationships

Consumer behaviour is complex and influenced by multiple factors simultaneously. Linear regression enables us to assess the effect of a single variable (such as gender) while controlling for the influence of other variables (such as age, income, and education). This allows us to isolate the specific effect of the variable of interest. The results of these analyses are presented in our regression Tables 4, 5, and 7, 8, 9, 10, 11. Columns 1 and 2 display results separately for the UK and the Netherlands, respectively. Each table reports the following:

- Coefficients, which indicate the size and direction of a variable's effect. For example, in Table 4, Column 1, the coefficient for 'Female' is -0.072. This means that, in the UK, being female is associated with a 0.072-point decrease in the adherence to the legal right score compared to men, after controlling for age and education.
- Standard errors, shown in parentheses beneath the coefficients, indicate how precise the estimate is. A smaller standard error suggests greater confidence in the coefficient's value.
- Significance stars, which provide a visual summary of statistical significance of a coefficient, based on the *p*-value. *** indicates *p* <.01 (1%; highly significant), ** indicates *p* <.05 (statistically significant), and * indicates *p* <.1 (marginally significant). Coefficients without stars are not statistically significant, meaning we cannot be confident that the factor has a meaningful effect.

### Testing for Cross-Country Differences (Interaction Effects)

A key question for our study is: does the effect of gender vary depending on the country? To address this, Column 3 in all tables extends the regression analysis by pooling data from both countries and adding an interaction term to the model. This term is created by multiplying two variables together (e.g., Female × Netherlands). A statistically significant interaction term provides evidence that the effect of one variable depends on the level of another. This approach allows us to move beyond simple statements such as “women differ from men” to more nuanced conclusions like “the way in which women differ from men is not the same in the UK as in the Netherlands.” To interpret a model with an interaction term, the following guidance applies:

To account for country-gender effects, the model includes four variables. To control for country, we include the indicator variable Netherlands (= 1; UK = 0). To control for gender, we include the indicator variable 'Female' (= 1; male = 0). The interaction term is the product of these two variables together, 'Female x Netherlands'. A baseline group must be defined, against which all other groups are compared. In our analysis, the baseline group is ‘men in the UK’, and is omitted from the model by coding 'Male x UK' as 0.

To interpret the coefficient effect sizes, we use the “Costs” model in Table 8 as an example:

- The coefficient for Female (0.072) now represents the effect of gender only within the UK, the baseline country. Thus, in the UK, women consider costs to be more important than men.
- The coefficient for the Netherlands (-0.237) represents the effect of country only for men, the baseline gender (Male). That is, Dutch men find costs less important than UK men.
- The Female × Netherlands interaction term (-0.154) is the key variable. It reflects the additional effect for individuals who are both female and Dutch. In other words, it captures the difference in the gender gap between the two countries. The negative sign indicates that the positive gender gap observed in the UK is significantly reduced, or possibly reversed, in the Netherlands.

This analysis demonstrates that the influence of gender on cost sensitivity differs fundamentally between the two countries. While women in the UK are significantly more sensitive to costs than UK men, this pattern does not hold for Dutch women and men. This is a critical insight that would not be revealed through a simpler analysis.

### A Note on Interpreting Effect Sizes

Throughout our analysis, we present measures that reflect the magnitude of effects, not only their statistical significance. For the bivariate comparisons in Table 6, we report Cohen's *d*; for the regression results, we convert coefficients into percentage changes.

Cohen's *d* expresses the difference between two group means in units of pooled standard deviation. It is calculated as the difference between the female and male means divided by the pooled standard deviation of the two groups, so a value of 0.20 means the two groups differ by one-fifth of a standard deviation. The sign indicates direction (in Table 6, a negative value means women score higher than men, a positive value means men score higher). By widely used convention, |*d*| ≈ 0.2 is considered a small effect, 0.5 a medium effect, and 0.8 a large effect. Cohen's *d* is useful because, unlike the raw difference in means, it is independent of the measurement scale and therefore comparable across the different items in Table 6. In our data, almost all of the gender differences fall below the threshold for a small effect, indicating that the differences, while often statistically significant, are modest in magnitude.

To convert regression coefficients into meaningful percentage changes, we use two approaches:

- Percentage of scale range: For variables measured on a scale with a defined range (e.g., a 1–5 scale has a range of 4), we calculate: coefficient ÷ scale range × 100. For example, a coefficient of 0.078 on a 1–5 scale represents 0.078 ÷ 4 = 1.95% of the total scale.
- Percentage relative to baseline prediction: We calculate: coefficient ÷ baseline predicted value × 100, where the baseline is the predicted score for the reference group (typically men in the UK). For example, with a coefficient of 0.078 and a baseline predicted score of 3.805, the calculation is 0.078 ÷ 3.805 × 100 = 2.05%, meaning the predicted score is approximately 2% higher than the baseline.

In our results, we primarily report the second approach, percentage relative to baseline, as it provides a more intuitive understanding of practical significance. For instance, when we state that UK women show “2% higher importance,” this indicates that their predicted score is 2% above the baseline prediction for UK men.

We apply this framework to various dependent variables:

- Adherence to the Legal Right Index (0–2 scale): A coefficient of -0.072 represents -0.072 ÷ 2 × 100 = -3.6% of the scale range. In practical terms, if men score an average of 1.03 correct answers, women score an average of 0.958 correct answers (or 7% lower relative to the baseline, -0.072 ÷ 1.03).
- Adherence to Procedural Conditions Index (0–6 scale): A coefficient of 0.187 represents 0.187 ÷ 6 × 100 = 3.1% of the scale range, indicating that women correctly identify about 0.187 more conditions than men.
- Factor Scales (1–5): A coefficient of 0.072 represents 0.072 ÷ 4 × 100 = 1.8% of the scale range. For percentage relative to baseline: if the baseline predicted value is 3.805, then 0.072 ÷ 3.805 × 100 = 1.9% higher than baseline.
- Enforcement Willingness (0–1 binary): A coefficient of -0.039 implies that women are 3.9 percentage points less likely to pursue enforcement than men.

Because our primary interest lies in gender differences, we focus on gender effects within countries. However, cross-country variation is also controlled for through our multivariate regression models.

### Robustness of the Procedural-Conditions Index

Tables 13 and 14 report the robustness check described in the Results, re-estimating the adherence to procedural conditions index on the "prove X" items only (Table 13) and the "pay X" items only (Table 14).
